# Effects of seed morphology and elaiosome chemical composition on attractiveness of five *Trillium* species to seed‐dispersing ants

**DOI:** 10.1002/ece3.6101

**Published:** 2020-02-27

**Authors:** Chelsea N. Miller, Susan R. Whitehead, Charles Kwit

**Affiliations:** ^1^ Department of Ecology and Evolutionary Biology University of Tennessee Knoxville TN USA; ^2^ Department of Biological Sciences Virginia Tech University Blacksburg VA USA; ^3^ Department of Forestry, Wildlife and Fisheries University of Tennessee Knoxville TN USA

**Keywords:** ants, chemical ecology, insect‐plant interaction, myrmecochory, oleic acid

## Abstract

Morphological and chemical attributes of diaspores in myrmecochorous plants have been shown to affect seed dispersal by ants, but the relative importance of these attributes in determining seed attractiveness and dispersal success is poorly understood. We explored whether differences in diaspore morphology, elaiosome fatty acids, or elaiosome phytochemical profiles explain the differential attractiveness of five species in the genus *Trillium* to eastern North American forest ants. Species were ranked from least to most attractive based on empirically‐derived seed dispersal probabilities in our study system, and we compared diaspore traits to test our hypotheses that more attractive species will have larger diaspores, greater concentrations of elaiosome fatty acids, and distinct elaiosome phytochemistry compared to the less attractive species. Diaspore length, width, mass, and elaiosome length were significantly greater in the more attractive species. Using gas chromatography–mass spectrometry, we found significantly higher concentrations of oleic, linoleic, hexadecenoic, stearic, palmitoleic, and total fatty acids in elaiosomes of the more attractive species. Multivariate assessments revealed that elaiosome phytochemical profiles, identified through liquid chromatography–mass spectrometry, were more homogeneous for the more attractive species. Random forest classification models (RFCM) identified several elaiosome phytochemicals that differed significantly among species. Random forest regression models revealed that some of the compounds identified by RFCM, including methylhistidine (*α*‐amino acid) and d‐glucarate (carbohydrate), were positively related to seed dispersal probabilities, while others, including salicylate (salicylic acid) and citrulline (*L‐α‐*amino acid), were negatively related. These results supported our hypotheses that the more attractive species of *Trillium*—which are geographically widespread compared to their less attractive, endemic congeners—are characterized by larger diaspores, greater concentrations of fatty acids, and distinct elaiosome phytochemistry. Further advances in our understanding of seed dispersal effectiveness in myrmecochorous systems will benefit from a portrayal of dispersal unit chemical and physical traits, and their combined responses to selection pressures.

## Graphical abstract

To investigate seed attributes that might impact attractiveness to ant dispersers, diaspore morphology, elaiosome phytochemistry, and elaiosome fatty acid profiles of five eastern North American species of *Trillium* that vary in their level of attractiveness to ant dispersers were assessed and compared. Seed attractiveness, measured as dispersal probability, was found to be related to broad interspecific differences in composition of elaiosome phytochemistry, to high concentrations of elaiosome fatty acids, and to larger diaspore width, length, mass, and elaiosome length. Further advances in our understanding of seed dispersal in myrmecochorous systems will require a portrayal of dispersal‐unit chemical and physical traits, and their combined responses to selection pressures.

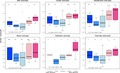

## 1 INTRODUCTION

The frequency and quality of seed dispersal by ants in the mutualism known as myrmecochory is influenced by a number of plant and disperser attributes. From the perspective of plants, traits characterizing the diaspore—the dispersal unit of a myrmecochorous plant, comprised of the seed and the elaiosome (a fleshy seed‐coat appendage rich in lipids, amino acids, and monosaccharides; Boieiro, Espadaler, Gómez, & Eustaquio, 2012; Brew, O'Dowd, & Rae, 1989; Fischer, Richter, Hadacek, & Mayer, 2008)—influence seed dispersal effectiveness (Schupp, 1993; Schupp, Jordano, & Gómez, 2010). Present in all myrmecochore species (Lisci, Bianchini, & Pacini, 1996), elaiosomes attract ants to pick up and carry diaspores back to the nest (Van der Pijl, 1982); much work has documented this for beneficial and effective seed‐dispersing ants such as those in the genera *Aphaenogaster* (Asia and North America; Ness, Morin, & Giladi, 2009; Takahashi & Itino, 2015), *Rhytidoponera* (Australia; Gove, Majer, & Dunn, 2007), *Myrmica* and *Formica* (Europe; Gorb & Gorb, 1995; Manzaneda & Rey, 2009).

Diaspore size, elaiosome biomass, and the elaiosome‐seed mass ratio are all known to influence the attractiveness of diaspores to ants (Leal, Lima Neto, Oliveira, Andersen, & Leal, 2014; Mark & Olesen, 1995). Interspecific variation in elaiosome size and elaiosome‐seed mass ratios is substantial, and these traits are often correlated (Edwards, Dunlop, & Rodgerson, 2006; Leal et al., 2014; Levine, Ben‐Zevi, Seifan, & Giladi, 2019; Reifenrath, Becker, & Poethke, 2012), meaning any interpretation of the effects of one trait should be considered in light of the other. In many systems, effective ant dispersers prefer the seeds of species that produce heavier elaiosomes (Levine et al., 2019; Ness, Bronstein, Andersen, & Holland, 2004; Takahashi & Itino, 2015), although Manzaneda, Rey, and Alcántara (2009) found that ants exerted significant selection on seed size, but not on elaiosome size when both traits were considered simultaneously. The nutritional quality of elaiosomes also likely plays a role in ant preference (Caut, Jowers, Cerdá, & Boulay, 2013; Fischer et al., 2008), given that ants disassemble the diaspore and feed the elaiosome to their larvae upon return to the nest (Fischer, Ölzant, Wanek, & Mayer, 2005). However, diaspore morphology and elaiosome nutritional rewards may not be the most important determinants of the first, critical step of the dispersal process: diaspore retrieval and dispersal to the nest. Instead, ant foraging behavior is strongly influenced by non‐nutritive elaiosome chemical cues (Nelson et al., 2019).

Elaiosome lipid chemistry has been well described (Boulay, Coll‐Toledano, & Cerdá, 2006; Boulay, Coll‐Toledano, Manzaneda, & Cerdá, 2007; Bresinsky, 1963; Brew et al., 1989; Fischer et al., 2008; Gammans, Bullock, Gibbons, & Schönrogge, 2006; Hughes & Westoby, 1994; Kusmenoglu, Rockwood, & Gretz, 1989; Lanza, Schmitt, & Awad, 1992; Leal et al., 2014; Lisci et al., 1996; Skidmore & Heithaus, 1988; Wu, Peng, Dong, Xia, & Zhao, 2019) and is known to influence both the quantitative and qualitative aspects of seed dispersal effectiveness (sensu Schupp, 1993; Schupp et al., 2010) by ants. Seed‐dispersing ants display a preference for food items containing high concentrations of oleic acid, an important signaling compound that stimulates foraging behavior, and thus select for seeds containing high concentrations of this compound (Boulay et al., 2006, 2007; Brew et al., 1989; Fischer et al., 2008; Lanza et al., 1992; Pfeiffer, Huttenlocher, & Ayasse, 2010; Turner & Frederickson, 2013). Other fatty acids, including palmitic acid, stearic acid, linoleic acid, and linolenic acid may also be attractive to ants and help initiate seed dispersal (O'Dowd & Hay, 1980).

Nonlipid chemical cues stemming from the broader phytochemical profiles of elaiosomes may also govern ant preference in myrmecochorous systems. Nonlipid elaiosome phytochemistry likely includes diverse nutritional rewards in addition to other plant primary and secondary metabolites. Such compounds may function to mediate interactions between myrmecochore seeds and other species in their environment, including mutualistic partners and antagonists such as granivores or pathogenic microbes. For example, capsaicin, a secondary metabolite responsible for heat in chili (*Capsicum,* Solanaceae) fruits, selectively deters vertebrate predators and microbial pathogens without deterring effective seed‐dispersing birds (Fricke, Haak, Levey, & Tewksbury, 2016; Tewksbury & Nabhan, 2001; Tewksbury et al., 2008); it is possible that secondary metabolites present in elaiosomes have similar mediating effects for myrmecochorous plants. Although we do not know which nonlipid phytochemical compounds are important to ant seed dispersers, we do know that ants utilize chemical reception and therefore could be affected by a suite of seed phytochemicals (Nelson et al., 2019).

Because elaiosome chemistry is under selection by ant dispersers (Boulay et al., 2006, 2007), it is possible that ants exert stabilizing selection on the entire phytochemical profile. Such stabilizing selection on the chemotype of plant‐derived food bodies by ant mutualists has been shown for myrmecophytic symbioses in the tropics (see the review by Mayer, Frederickson, McKey, & Blatrix, 2014). Extrafloral nectaries (EFN) and pearl bodies produced by myrmecophytic plants are rich in carbohydrates/amino acids and lipids, respectively (Bluthgen, Gottsberger, & Fiedler, 2004; Buono, Oliveira, & Sousa Paiva, 2008; Fischer, Richter, Wanek, & Mayer, 2002; Gonzalez‐Teuber & Heil, 2009; Heil, 2011; Heil, Baumann, Kruger, & Linsenmair, 2004; Heil, Fiala, Kaiser, & Linsenmair, 1998; Paiva, Buono, & Lombardi, 2009; Rickson, 1976; Shenoy, Radhika, Satish, & Borges, 2012; Webber, Abaloz, & Woodrow, 2007), and these food resources are typically not as attractive to generalist ants as they are to the plants' specific ant mutualists (Bluthgen & Fiedler, 2004; Davidson, Foster, Snelling, & Lozada, 1991; Gonzalez‐Teuber & Heil, 2009; Heil et al., 2004, 1998). Furthermore, ant preferences for specific chemical compounds in EFN are highly species‐specific, suggesting mutualists exert strong stabilizing selection on EFN chemotypes. Although the myrmecochory is a facultative mutualism rather than an obligate mutualism as in tropical myrmecophytic systems, it is possible that seed‐dispersing ants nevertheless exercise a similar stabilizing selection on elaiosome phytochemical profiles such that a certain narrow chemotype is defined. This hypothesis has not been tested in myrmecochorous systems; in particular, studies are lacking that characterize the metabolomic profiles of elaiosome phytochemistry and assess whether single compounds or entire phytochemical profiles affect diaspore retrieval and dispersal to the nest by ants.

Despite the fact that three main aspects of diaspores (morphological, nutritional, chemical) have been linked to seed dispersal by ants, rarely have multiple diaspore traits been investigated in the same study (but see Leal et al., 2014), and the most important mechanisms governing ant preference remain unclear. We explore whether differences in diaspore morphology, elaiosome fatty acids, or elaiosome phytochemical profiles explain the differential attractiveness of diaspores of plants in the genus *Trillium* (Order Liliales, Family Melanthiaceae) to ants, primarily of the keystone seed‐dispersing genus *Aphaenogaster.* Although a number of ant species disperse seeds of myrmecochores in eastern North America (Gaddy, 1986; Zelikova, Sanders, & Dunn, 2011), *Aphaenogaster* ants are the primary seed dispersal vector for most temperate ant‐dispersed flora in this region and are responsible for approximately 74% of myrmecochore seed collection in forests where *Aphaenogaster* have been reported (Ness et al., 2009). Our study system is comprised of five eastern North American *Trillium* species, two of which are geographically widespread and three of which are narrowly endemic; herein, pairs of widespread and endemic species co‐occur in multiple sites across the southern Appalachian region of the United States. In this system, *Aphaenogaster* ants display a preference for dispersing the diaspores of the geographically widespread trilliums compared to their co‐occurring, endemic congeners (Miller & Kwit, 2018). Using field‐based observations of seed dispersal probabilities from Miller and Kwit (2018), we ranked each of the five study species from least attractive to most attractive (see Materials and Methods below) and use this scale of attractiveness as a comparative framework for exploring diaspore morphology and elaiosome chemistry throughout the present study.

Our first objective is to quantify interspecific differences in diaspore morphology among our study species. We predict that the more attractive species of *Trillium* will have larger diaspores overall, and higher elaiosome mass and elaiosome‐seed mass ratios than less attractive species. Our second objective is to assess interspecific differences in concentrations of elaiosome fatty acids. We predict that concentrations of key fatty acids, including oleic acid, will be higher in the elaiosomes of the more attractive species. Our third objective is to assess interspecific differences in elaiosome phytochemical profiles. We predict that elaiosome phytochemical profiles of the more attractive species will be more tightly clustered in multivariate space than those of less attractive species, reflecting greater stabilizing selection by ant dispersers.

## 2 MATERIALS AND METHODS

### 2.1 Study species and sites

Eastern North America is a biodiversity hotspot for the plants in the genus *Trillium—*a group of myrmecochorous (ant seed dispersed) perennial understory forest herbs*—*with at least 29 species occurring in this region (Freeman, 1975; Ohara, 1989). In the southern Appalachian region, many species of *Trillium* are sympatric. Although co‐occurring myrmecochore species often temporally stagger fruiting (Gordon, Meadley‐Dunphy, Prior, & Frederickson, 2019; Warren, Giladi, & Bradford, 2014), the sympatric species studied here have overlapping flowering and fruiting phenology (Miller & Kwit, 2018). Due to the spatial proximity of plants at our study sites, foraging ants occasionally come across mature diaspores of co‐occurring congeners at the same time (C. N. M., personal observation), resulting in potential interspecific competition for dispersal services. We investigated the importance of morphological and chemical diaspore attributes in determining seed attractiveness to foraging ants using the five species of *Trillium* studied in Miller and Kwit (2018). These included the geographically widespread species *Trillium catesbaei* Elliott and *T. cuneatum* Raf., as well as the range‐restricted, endemic species *T. lancifolium* Raf., *T. discolor* Wray ex Hook, and *T. decumbens* Harbison. Pairs of these species co‐occur in multiple locations throughout the southern Appalachians (see Miller & Kwit, 2018).

We located six study sites in spatially distinct forest stands (average size of 130 ha; stands separated from one another by at least 3 km) containing sympatric populations of species pairs, and one additional site containing only *T. lancifolium* to supplement a lack of available mature fruits for this species at the other sites (*n* = 7 sites; Table 1). Five sites were located in northwest Georgia, and two sites were located near the borders of Georgia, North Carolina, and South Carolina. All sites were low‐lying, mesic, deciduous forests with moderate to thick canopy cover. Sites in northwest Georgia were located in the Limestone Valley soil province, and sites at the Georgia‐North Carolina‐South Carolina borders were located in the Blue Ridge soil province; both provinces are characterized by mildly acidic soils high in phosphate. Sites in northwest Georgia were dominated by tulip poplar (*Liriodendron tulipifera*), American beech (*Fagus grandiflora*), and oak species (*Quercus* spp.). Sites at the Georgia‐North Carolina‐South Carolina borders were characterized by tulip poplar, American holly (*Ilex opaca*), and eastern hemlock (*Tsuga canadensis*). All sites had understory layers characterized by red buckeye (*Aesculus pavia*), goldenseal (*Hydrastis canadensis*), jack‐in‐the‐pulpit (*Arisaeama triphyllum*), and numerous herbaceous myrmecochorous species including trout lily (*Erythronium americanum*), violets (*Viola* spp.), bloodroot (*Sanguinaria canadensis*), wood anemone (*Anemone quinquefolia*), and sharp‐lobed hepatica (*Anemone acutiloba*).

**Table 1:** Study species collected at field sites in June 2018, and their relative status as attractive or less attractive to ant dispersers

Species	Status	Site	State
*T. cuneatum*	Attractive	Cave	Georgia
*T. cuneatum*	Attractive	Pocket branch	Georgia
*T. cuneatum*	Attractive	Old mine	Georgia
*T. cuneatum*	Attractive	Blue hole	Georgia
*T. catesbaei*	Attractive	Jocassee Gorges	South Carolina
*T. catesbaei*	Attractive	Whitewater falls	North Carolina
*T. decumbens*	Less Attractive	Cave	Georgia
*T. decumbens*	Less Attractive	Pocket branch	Georgia
*T. discolor*	Less Attractive	Jocassee Gorges	South Carolina
*T. discolor*	Less Attractive	Whitewater falls	North Carolina
*T. lancifolium*	Less Attractive	Tilton bridge	Georgia
*T. lancifolium*	Less Attractive	Blue hole	Georgia

**Figure 1:**
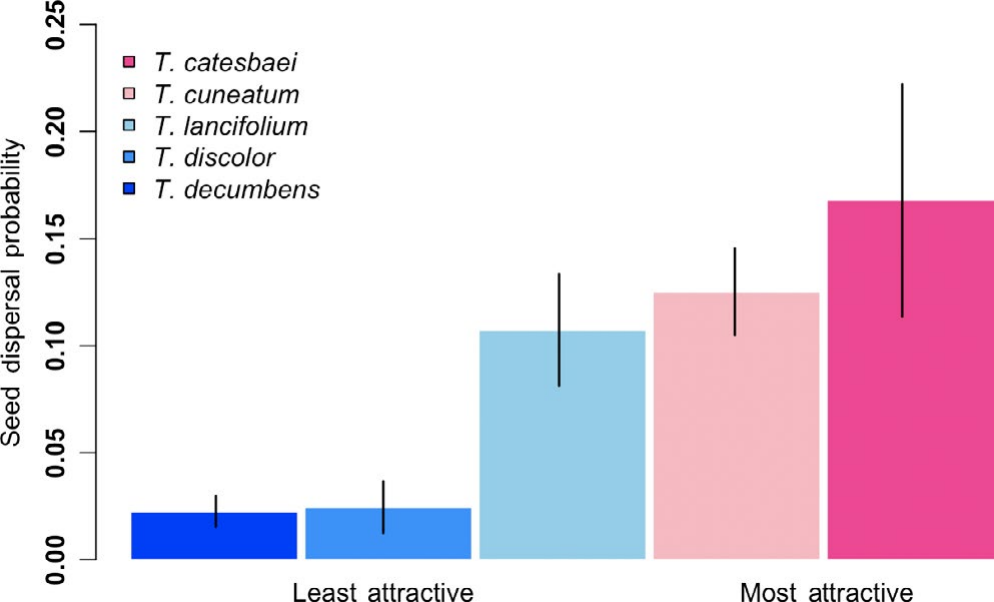
Empirically‐derived averaged probability of seed dispersal by ants, primarily of the genus *Aphaenogaster,* for five species of *Trillium* at seven sites in the southeastern U.S. Bars represent standard error. Blue indicates lower seed dispersal probabilities (i.e., “least attractive” species) while pink indicates highest seed dispersal probabilities (i.e., “most attractive” species)

We collected mature fruits just prior to natural dehiscence from the study species at their respective sites during summer 2018 for use in morphological and chemical analyses. Upon return to the laboratory, we stored fruits at −20°C for 1 month prior to diaspore morphological analyses and 4 months prior to chemical analyses. To organize the species by their overall attractiveness to ants, we used seed dispersal probabilities calculated for each species based on in situ observations conducted during a previous study in the same study system (Miller & Kwit, 2018). In that study, the proportion of seeds removed by ants (primarily of the genus *Aphaenogaster*) in 1 hr from natural seed depots was averaged for 15 individuals of each species at three study sites; the proportion across sites for each species was then averaged (*n* = 45 hr of observation/species). We interpret these averaged proportions as the probability of seed dispersal, a measure of attractiveness for each species of *Trillium* and a comparative framework for the present study*. Trillium catesbaei* had the highest average proportion of seeds removed at 16.8% (“most attractive”) followed by *T. cuneatum* at 12.5%; *T. lancifolium* at 10.7%; *T. discolor* at 2.5%, and *T. decumbens* at 2.2% (“least attractive”; Figure 1).

### 2.2 Diaspore morphology

To address our first study objective, quantifying interspecific differences in diaspore morphology, we measured diaspore and elaiosome length, width, and mass for 26 diaspores of each species, representing multiple individuals from each study site (*n* = 130). We took the fresh mass of entire diaspores (g) and then measured the length and width of diaspores (mm) using digital calipers (Mitutoyo Digimatic Caliper, 0.01 mm resolution). We then removed the elaiosome from each seed using a straight razor and repeated the above measurements for the elaiosome. Elaiosome‐seed mass ratios were calculated by dividing the mass of the elaiosome by the mass of the entire diaspore.

After evaluating each response variable (diaspore length, diaspore width, diaspore mass, elaiosome length, elaiosome width, elaiosome mass, and elaiosome‐seed mass ratio) for normality by generating normal probability (Q‐Q) plots and histograms to visualize model residuals, we determined that the residuals of all response variables were normally distributed. Each response variable was then compared for the five study species using a linear mixed‐effects model in the package lmerTest (Kuznetsova, Brockhoff, & Christensen, 2017) in R (R Core Team, 2017). This test uses the Satterthwaite's method for approximating degrees of freedom for *t* and *F* tests, which is more conservative than residual degrees of freedom approximations; therefore, the assumption of homogeneity of variances across samples can be relaxed (Keselman, Algina, Kowalchuk, & Wolfinger, 1999). Each model included one morphological trait as the response variable, species of *Trillium* as the fixed effect, and study site as a random effect to account for the potential effect of environmental heterogeneity or intraspecific population. Tukey post hoc tests were performed to identify the pairwise direction of effects using the package multcomp (Hothorn, Bretz, & Westfall, 2008). To visualize trends, which were impacted by the random effect of study site, we generated model residuals from linear models with the effect of site partialled out. Boxplots display these model residuals, which depict the isolated effect of species on diaspore morphology traits.

### 2.3 Elaiosome fatty acid profiles

To address our second study objective, quantifying interspecific differences in concentrations of elaiosome fatty acids among species of *Trillium*, we assessed triglyceride, diglyceride, and free fatty acid forms using gas chromatography–mass spectrometry (GC‐MS). Detailed methodology relating to the GC‐MS analysis can be found in Data S1. We produced standard curves by running five concentrations (10, 1, 0.5, 0.1, 0.05, and 0.025 mg/ml) of a fatty acid methyl ester standard mixture of methyl linoleate (20 wt%), methyl linolenate (20 wt%), methyl oleate (20 wt%), methyl palmitate (20 wt%), and methyl stearate (20 wt%) (CRM1891, Millipore Sigma, Merck KGaA). Linearity across the relevant range of concentrations was evaluated visually and by calculating the slope of the line and *R* ^2^ values for each compound. GC‐MS produced TIC chromatograms for each elaiosome fraction (free, di‐, and triglycerides). Peaks were identified based on comparisons of retention times and spectra with authentic standards. Compounds for which we did not have standards were tentatively identified using the NIST 14 library (Scientific Instrument Services). Peak areas were determined using the Integrator Trapezoid feature in OpenChrom (Wenig & Odermatt, 2010) and concentrations were determined for known compounds based on response factors of standards relative to the internal standard. Concentrations for tentatively identified compounds were estimated as internal standard equivalents. All concentrations were converted to a percentage of elaiosome fresh weight for final reporting and statistical analyses.

Total fatty acid concentrations and concentrations of fatty acids in free, di‐, and triglyceride forms were compared for the five study species using linear mixed‐effects models in the package lmerTest in R, as above. Concentrations (% fresh weight) of five individual fatty acids and of total fatty acids were evaluated for normality prior to running linear mixed‐effects models; in each case, the assumption of normality was violated. Therefore, we applied data transformations to raw concentrations to approximate normality (logarithmic or square‐root; see Keene, 1995; Osborne, 2002). As in the *Diaspore Morphology* methods above, our use of the Satterthwaite's method for approximating degrees of freedom allowed us to relax the assumption of homogeneity of variances. Study site was included as a random effect in each model to account for the effects of environmental heterogeneity or interspecific population. Species and fatty acid form (free, di‐, or triglyceride) were included as fixed effects. Interactions between species and form were tested for each model; in no case was the interaction term significant, so interactions were dropped from all models. Tukey post hoc tests were performed to identify the pairwise direction of effects between species and between fatty acid forms. To visualize trends, we generated boxplots using model residuals as in the *Diaspore Morphology* methods above.

### 2.4 Elaiosome phytochemical profiles

To address our third study objective, assessing interspecific differences in elaiosome phytochemistry, we characterized and compared profiles of both known and unknown elaiosome metabolites using liquid chromatography–mass spectrometry (LC‐MS). Each of the five study species was represented by six sample replicates (*N* = 30). Samples were taken from 2 to 6 individuals from the representative study sites (Table 1). Detailed methodology relating to the LC‐MS analysis can be found in Data S1. LC‐MS produced relative concentrations of phytochemical compounds across samples, which were compared using z‐transformed peak areas from extracted ion chromatograms.

To visualize clustering of elaiosome phytochemical profiles across species, we performed a constrained redundancy analysis (RDA) using the vegan package (Oksanen et al., 2017) in R with species of *Trillium* as the explanatory variable and peak areas of the partial phytochemical data (known compounds; *n* = 122) as the response. RDA, a method to summarize the variation in a set of response variables that can be explained by a set of explanatory variables, is a constrained version of principal components analysis wherein canonical axes (i.e., linear combinations of response variables) must also be linear combinations of the explanatory variables (Legendre & Legendre, 1998). We did not explicitly account for variation in the phytochemistry data that may have been explained by study site because RDA partitions the total variance of the data into constrained variances (i.e., variation in the response matrix that is redundant with the variation in the explanatory matrix) and unconstrained variances (i.e., variation in the response matrix that is not redundant with the variation in the explanatory matrix), which can be compared to determine how much of the variation in the response is accounted for by the explanatory variable (Legendre & Legendre, 1998). We used all six sample replicates per species, for a total of 30 samples. The model ran for 1,000 permutations and the significance of the explanatory variables and the first two RDA axes were assessed using ANOVA. We repeated this procedure for the full phytochemical data (known compounds + unknown features; *n* = 7,552) detected by LC‐MS.

To better understand the major chemical drivers of the multivariate spread of the partial (i.e., known compounds) and full (i.e., known + unknown compounds) phytochemical data, we constructed four random forest models using the package randomForest (Liaw & Wiener, 2002) in R. Random forest is a supervised machine learning technique that builds a classification tree by repeatedly splitting the data based on whether or not they fall above or below a threshold value of each explanatory variable in the model (Biau, 2012; Bielby, Cardillo, Cooper, & Purvis, 2010). Random forest ranks the relative importance of different predictors in distinguishing among the levels and provides a measure of prediction accuracy, cross‐validation, for correctly classifying unknown samples into groups according to the predictor variables. By training the model using a subset of samples, random forest is able to classify the remaining samples with higher accuracy than would be achieved by always guessing the most common category.

In our analysis, random forest classification models (RFCMs) were implemented to identify compounds with the highest relative importance in distinguishing among the five species of *Trillium,* a categorical level. Two RFCMs were used evaluate the partial and full phytochemical data sets, respectively. Peak areas of the known compounds were used as the set of possible predictors for the partial RFCM (*n* = 122), whereas peak areas of all compounds (known + unknown) were used as the set of possible predictors for the full RFCM (*n* = 7,552). Following implementation of the RFCMs, two random forest regression models (RFRMs) were used to identify compounds with the highest relative importance in distinguishing among the average probability of seed dispersal for each species, a continuous level (16.8% ‐ *T. catesbaei*, 12.5% ‐ *T. cuneatum*, 10.7% ‐ *T. lancifolium*, 2.5% ‐ *T. discolor*, and 2.2% ‐ *T. decumbens*). Peak areas of the known compounds were used as predictors for the partial RFRM, and peak areas of all compounds were used as the set of predictors for the full RFRM, as in the RFCMs above. Boruta variable selection was used in all four models to select relevant compounds associated with the levels (i.e., *Trillium* species in RFCMs, and seed dispersal probabilities in RFRMs). Unlike regular random forest variable selection procedures, which choose only the smallest number of variables that allow accurate classification of unknown samples to groups, the Boruta algorithm compares the importance of each variable with a set of “shadow” variables created by randomly shuffling the values of the original variable to list all of the compounds that are likely to differ among groups (Kursa & Rudnicki, 2010). For the selected set of known candidate compounds in models, we assigned molecular formulas and compound classes using the ClassyFire software (Feunang et al., 2016). We followed RFCMs with a MANOVA and then separate ANOVAs, and RFRMs with linear regressions on each set of selected compounds. Predictors were considered significant at *α* = 0.05.

## 3 RESULTS

### 3.1 Diaspore morphology

**Figure 2:**
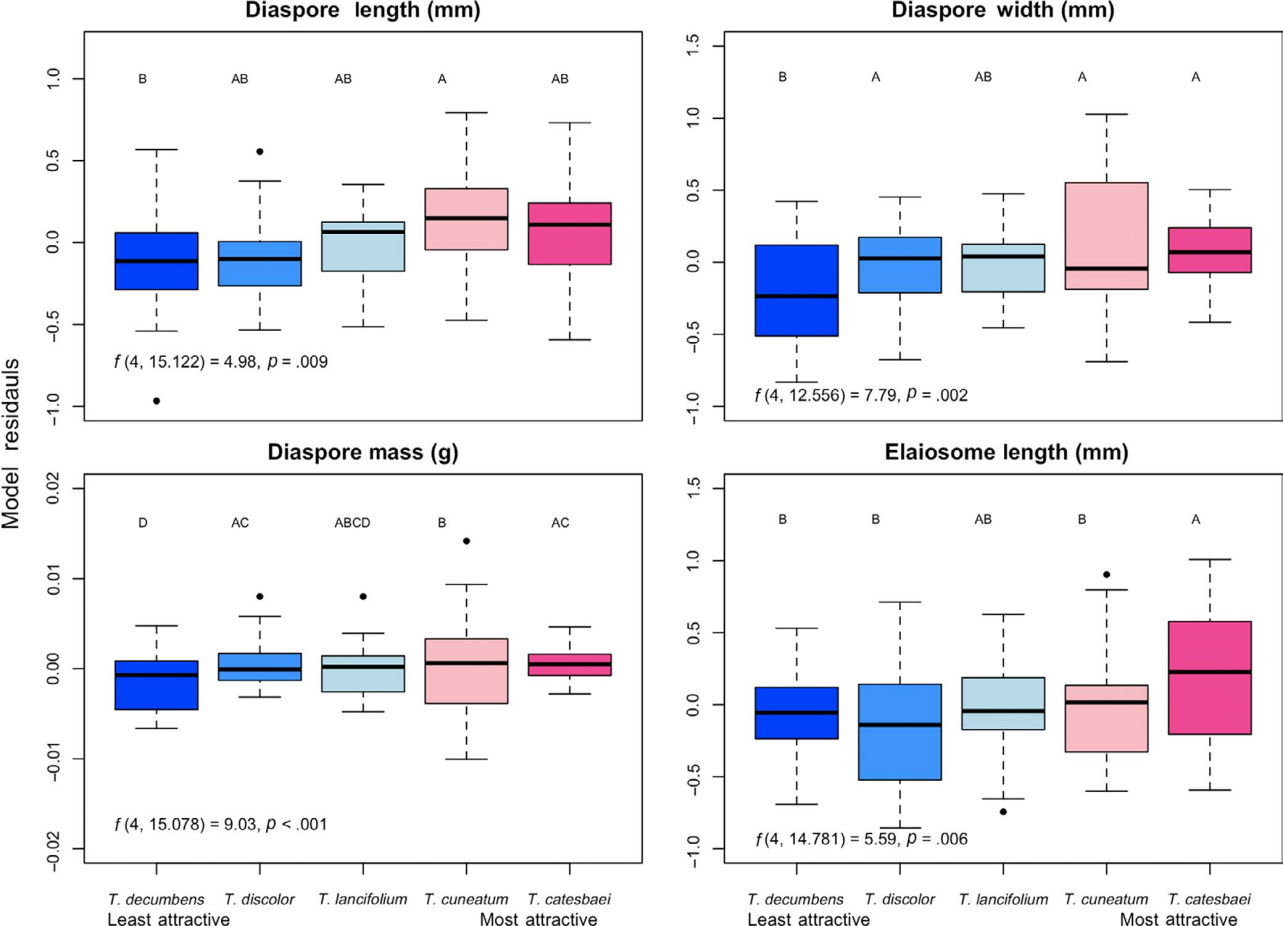
Median, interquartile range, and outliers for model residuals depicting the differences in diaspore length (top left), diaspore width (top right), diaspore mass (bottom left), and elaiosome length (bottom right) among the five species of *Trillium* (blue = least attractive species, pink = most attractive species), with the effects of site partialled out. Statistical results of linear mixed‐effects models are included at the bottom left of each plot. Letters indicate Tukey post hoc pairwise differences

Diaspore length, width, and mass differed significantly among the five *Trillium* species (*F_4,15_* = 4.98, *p* = .009; *F_4,13_* = 7.79, *p* = .002; *F_4,15_* = 9.03, *p* < .001, respectively). Elaiosome length also differed significantly among species (*F_4,15_* = 5.59, *p* = .006). Post hoc comparisons revealed that the second most attractive species, *T. cuneatum,* had significantly greater diaspore length than the least attractive species, *T. decumbens;* that the two most attractive species, *T. catesbaei* and *T. cuneatum,* had significantly greater diaspore width than the least attractive species; and that the two most attractive species had significantly greater diaspore mass than the least attractive species (Table S1; Figure 2). Post hoc comparisons revealed that the most attractive species, *T. catesbaei,* had significantly greater elaiosome length than all of the other species except for *T. lancifolium*. Elaiosome width, mass, and elaiosome‐seed mass ratio did not differ among species at the *α* = 0.05 threshold (*F_4,12_* = 0.92, *p* = .49; *F_4,12_* = 0.02, *p* = .99; *F_4,10_* = 2.33, *p* = .13; Table 2).

**Table 2:** Mean ± standard error for diaspore and elaiosome morphological metrics

Metric (mean +‐ S.E.)	*T. catesbaei*	*T. cuneatum*	*T. lancifolium*	*T. discolor*	*T. decumbens*
Diaspore mass (g)	0.012 ± 0.001	0.02 ± 0.001	0.02 ± 0.001	0.02 ± 0.002	0.02 ± 0.001
Diaspore width (mm)	2.16 ± 0.09	2.65 ± 0.09	2.30 ± 0.05	2.29 ± 0.11	2.25 ± 0.08
Diaspore length (mm)	3.35 ± 0.12	4.09 ± 0.07	3.52 ± 0.04	3.55 ± 0.15	3.63 ± 0.07
Elaiosome mass (g)	0.001 ± 0.0001	0.004 ± 0.0002	0.003 ± 0.0002	0.003 ± 0.0004	0.004 ± 0.0002
Elaiosome width (mm)	1.03 ± 0.06	1.73 ± 0.06	1.63 ± 0.06	1.39 ± 0.11	1.64 ± 0.08
Elaiosome length (mm)	2.62 ± 0.13	3.18 ± 0.08	2.86 ± 0.07	2.79 ± 0.20	2.92 ± 0.06
Elaiosome‐seed mass ratio (%)	0.14 ± 0.01	0.20 ± 0.02	0.19 ± 0.01	0.16 ± 0.02	0.23 ± 0.01

### 3.2 Elaiosome fatty acid profiles

**Figure 3:**
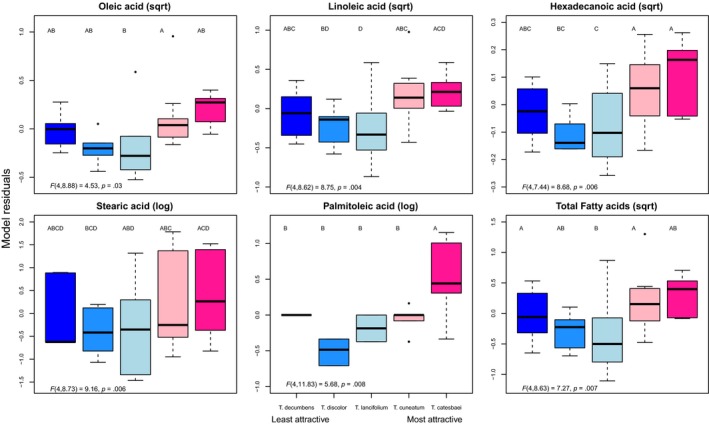
Median, interquartile range, and outliers for model residuals depicting the differences in concentrations (% fresh weight) of five individual fatty acids and total fatty acids among the five species of *Trillium* (blue = least attractive species, pink = most attractive species), with the effects of site partialled out. Raw values were transformed to approximate normality; transformation is indicated at the top of each plot. Statistical results of linear mixed‐effects models are included at the bottom left of each plot. Letters indicate Tukey post hoc pairwise differences

Gas chromatography–mass spectrometry identified a number of short‐chain fatty acids (≤ C18). The major compounds included oleic acid (C 18:1), linoleic acid (C 18:2), hexadecenoic acid (C 16:2), stearic acid (C 18:0), and palmitoleic acid (C 16:2); these were detected in all three forms (free, di‐, and triglycerides; Table 3). Concentrations of oleic, linoleic, hexadecenoic, stearic, palmitoleic, and total fatty acids differed significantly among the five *Trillium* species (*F_4,9_* = 4.53, *p* = .03; *F_4,9_* = 8.75, *p* = .004; *F_4,7_* = 8.68, *p* = .006; *F_4,9_* = 9, *p* = .006; *F_4,12_* = 5.68, *p* = .008; *F_4,9_* = 7.27, *p* = .007, respectively). Post hoc tests revealed that the most preferred species, *T. catesbaei,* had significantly greater concentrations of all compounds and of total fatty acids than the least preferred species, *T. decumbens* (Table S1; Figure 3). All raw data associated with this study are deposited in Dryad digital repository (d.o.i: https://doi.org/10.5061/dryad.hhmgqnkcz).

**Table 3:** Mean ± standard error of fatty acid concentrations across species (averaged across fatty acid forms; top)

*Trillium*	*cuneatum*		*catesbaei*		*lancifolium*		*discolor*		*decumbens*	
Compound	Mean	SE	Mean	SE	Mean	SE	Mean	SE	Mean	SE
Hexadecanoic acid	0.136	0.031	0.365	0.131	0.082	0.032	0.166	0.092	0.048	0.014
Oleic acid	0.624	0.230	1.456	0.687	0.420	0.246	0.721	0.480	0.128	0.047
Stearic acid	0.043	0.018	0.059	0.021	0.040	0.024	0.014	0.006	0.012	0.008
Palmitoleic acid	0.007	0.007	0.082	0.058	0.000	0.000	0.002	0.002	0.000	0.000
Linoleic acid	0.971	0.271	1.345	0.368	0.539	0.300	1.084	0.681	0.271	0.097
Total fatty acids	2.028	0.539	3.371	1.131	1.202	0.649	2.112	1.271	0.656	0.230

Form	Diglyceride		Triglyceride		Free	
Compound	Mean	SE	Mean	SE	Mean	SE
Hexadecanoic acid	0.054	0.016	0.256	0.082	0.157	0.032
Oleic acid	0.281	0.119	1.355	0.426	0.351	0.101
Stearic acid	0.003	0.002	0.024	0.01	0.079	0.017
Palmitoleic acid	0.002	0.002	0.044	0.031	0.004	0.003
Linoleic acid	0.304	0.105	1.503	0.374	0.783	0.223
Total fatty acids	0.78	0.271	3.281	0.869	1.637	0.359

Note

Mean ± standard error of fatty acid concentrations within fatty acid forms (bottom).

### 3.3 Elaiosome phytochemical profiles

**Figure 4:**
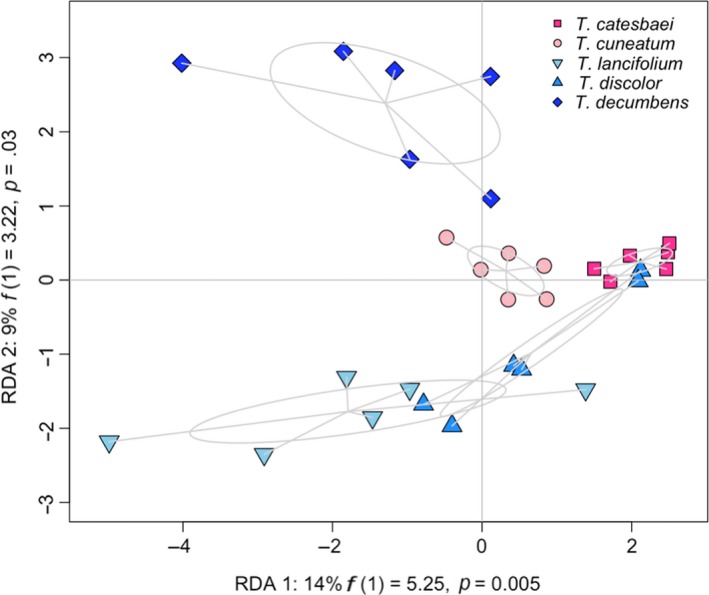
Constrained RDA evaluating general elaiosome phytochemical profiles (*n* = 6 samples per species; *n* = 30), with relative abundance of 122 known phytochemical compounds as the response and species as the fixed effect. Ellipses represent the standard deviation of the points around the centroid for each species. Blue = least attractive species, pink = most attractive species. The overall model is significant (*p* < .001), meaning species is a significant predictor of phytochemical composition of elaiosomes

Across the thirty elaiosome samples, 122 known compounds and 7,431 unknown features (paired *m/z* and retention times, which could correspond to individual compounds, adducts, or other artifacts; Clasquin, Melamud, & Rabinowitz, 2012) were detected by LC‐MS. The RDA for known phytochemical compounds was significant (*p* < .001; Figure 4), with 32.03% of the variation in the data explained by species of *Trillium*. The first and second constrained axes explained 14.28% and 8.76% of the variation respectively, and were both significant (*p* = .005; *p* = .029, respectively). The phytochemical profiles of the two most attractive species, *T. catesbaei* and *T. cuneatum,* did not overlap, but the spread of the data for both species was tightly constrained along the second axis. The RDA for the full phytochemical profiles of elaiosomes (including both the 122 known compounds and 7,431 unknown features) was significant (*p* = .03), with 15.52% of the variation in the data explained by the constrained RDA. Species of *Trillium* was thus maintained as a significant explanatory variable for elaiosome phytochemical profiles when considering the full set of LC‐MS results; this finding was further supported by the clear grouping of species in the full RDA (Figure S1).

**Figure 5:**
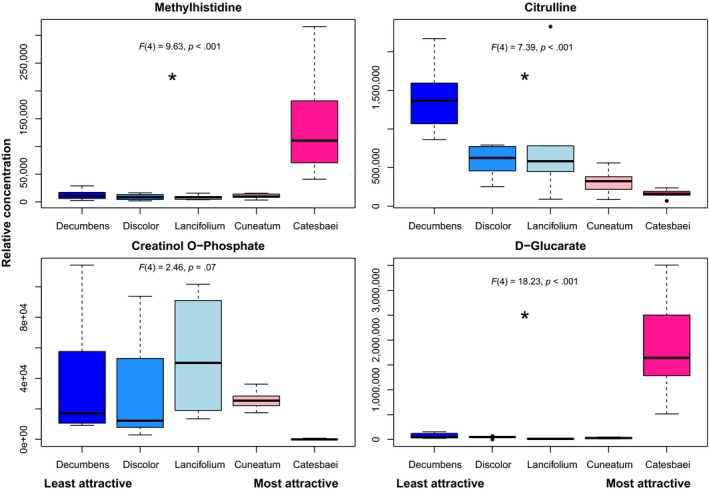
Median, interquartile range, and outliers for relative concentrations of four elaiosome phytochemicals selected by all random forest models as important in distinguishing among species of *Trillium* (blue = least attractive species, pink = most attractive species). Statistical results of follow‐up ANOVA models are included at the top of each plot

The composition of known phytochemicals in elaiosomes was distinguishable among species of *Trillium* based on the partial RFCM. The out‐of‐bag error rate was 0.10, meaning the model had 90% sample classification accuracy. Boruta variable selection identified 23 of the possible 122 compounds as important classifiers for species of *Trillium.* A MANOVA showed a significant overall difference among species for these 23 compounds (*F_20,96_* = 6.51, *p* < .001). Follow‐up ANOVAs revealed that 20 of the 23 compounds were significantly different among the five species (Table S2). The composition of known phytochemicals in elaiosomes was also distinguishable among averaged seed dispersal probabilities associated with each of the species of *Trillium* based on the partial RFRM. The model explained 57.27% of the variation in the data, and identified 12 of the possible 122 compounds as important in distinguishing among dispersal probabilities. Follow‐up regressions revealed that 8 of the 12 variables were significantly different among the average seed dispersal probabilities. Of the 12 compounds identified by the partial RFRM, 10 were also identified by the partial RFCM; these include salicylate, xanthine, histidine, 3, 4‐dihydroxyphenylacetate, methylhistidine, citrulline, glucosamine, creatinol o‐phosphate, d‐glucarate, and pantothenate (Table 4). Salicylate, xanthine, citrulline, glucosamine, and pantothenate had significant negative relationships with seed dispersal probability, while methylhistidine and d‐glucarate had significant positive relationships with dispersal probability (Table S2). Results of the full (known + unknown) RFCM and RFRM are included in Data S1. All four models identified methylhistidine, citrulline, creatinol o‐phosphate, and d‐glucarate as important (Figure S2). Follow‐up ANOVAs revealed significant differences in relative concentrations of three of the four compounds (methylhistidine, citrulline, and d‐glucarate) among the five study species (*F_4,8.93_* = 9.63, *p* < .001; *F_4,11.26_* = 7.39, *p* < .001; *F_4,12.87_* = 18.23, *p* < .001, respectively) and a marginally significant difference in relative concentration of creatinol o‐phosphate among the study species (F_4,8.98_ = 2.46, *p* = .07; Figure 5).

**Table 4:** Known nonlipid phytochemical compounds selected by both the partial RFCM and the partial RFRM and their chemical formulas and compound classes

Compound	Formula	Compound class
Salicylate	C_7_H_5_O_3_	Salicylic acids
Xanthine	C_5_H_4_N_4_O_2_	Purines
Histidine	C_6_H_9_N_3_O_2_	α ‐amino acids
3,4‐Dihydroxyphenylacetic acid	C_8_H_8_O_4_	Benzenediols
Methylhistidine^a^	C_7_H_11_N_3_O_2_	α ‐amino acids
Citrulline^a^	C_6_H_13_N_3_O_3_	*L*‐ α ‐amino acids
Glucosamine	C_6_H_13_NO_5_	Monosaccharides
Creatinol O‐Phosphate^a^	C_4_H_12_N_3_O_4_P	Phosphate esters
D‐Glucarate^a^	C_6_H_8_O_8_	Carbohydrates
Pantothenate	C_9_H_16_NO_5_	β‐amino acids

a Indicate the compounds selected by all four RF models.

## 4 DISCUSSION

In this study, we investigated the differences in diaspore morphology, elaiosome fatty acids, and elaiosome phytochemisty among five species of *Trillium* with different levels of attractiveness to seed‐dispersing ants in the southern Appalachian region of North America. Of the morphology metrics considered in our study, diaspore length, diaspore width, diaspore mass, and elaiosome length were significantly different among species, and post hoc tests revealed that values of these traits tended to increase with seed attractiveness. These findings provide support for the established hypothesis that seed size is a key trait determining the probability of seed dispersal (Manzaneda et al., 2009), with larger diaspores overall being preferred by seed‐dispersing ants (Hughes & Westoby, 1992; Takahashi & Itino, 2015).

Contrary to our prediction, elaiosome mass, elaiosome width, and elaiosome‐seed mass ratio were not significantly different among species. These results are not consistent with the results of several studies showing that ants prefer larger elaiosome biomass and/or elaiosome‐seed mass ratios (Leal et al., 2014; Levine et al., 2019; Mark & Olesen, 1995), although they do support the finding that ants do not exert significant selection on elaiosome size in *Helleborous foetidus* (Manzaneda et al., 2009). Considering that the most attractive species in our study, *T. catesbaei,* had smaller average elaiosome mass and smaller elaiosome‐seed mass ratios than all of its congeners, these aspects of elaiosome morphology do not appear to be the most important factors contributing to the attractiveness of *Trillium* seeds to ants. Larger diaspores likely enhance attractiveness of seeds to ants, but ants may not always prefer seed species with larger elaiosome mass or elaiosome‐seed mass ratios.

The results of the GC‐MS analysis indicate that elaiosome fatty acid composition is generally homogeneous across these species of *Trillium.* This result is supported by the conclusions of Fischer et al. (2008), who found that elaiosomes of myrmecochores in different families had homogeneous fatty acids dominated by oleic, palmitic, linoleic, and γ‐linolenic acids (see also: Boieiro et al., 2012; Boulay et al., 2006; Fischer et al., 2005; Hughes et al., 1994; Leal et al., 2014; Pfeiffer et al., 2010; Soukup & Holman, 1987). Despite the homogeneity of fatty acid composition, interspecific differences in concentrations of total and individual fatty acids appear to be associated with seed attractiveness to ants. Our prediction that the more attractive species of *Trillium* would contain higher total concentrations of fatty acids was supported, corroborating the majority of studies of elaiosome chemistry (Boieiro et al., 2012; Boulay et al., 2006; Brew et al., 1989; Fischer et al., 2008; Fokuhl, Heinze, & Poschlod, 2007; Gammans et al., 2006; Hughes & Westoby, 1994; Kusmenoglu et al., 1989; Lanza et al., 1992; Leal et al., 2014; Morrone, Vega, & Maier, 2000; Soukup & Holman, 1987; Wu et al., 2019).

The most attractive species also had significantly higher concentrations of oleic acid than the less attractive species, providing support for the hypothesis that this compound acts as a behavior‐releasing signal that stimulates ants to pick up and carry items to or from the nest (Brew et al., 1989; Marshall, Beattie, & Bollenbacher, 1979; Qiu et al., 2015; Skidmore & Heithaus, 1988). Oleic acid is the most abundant fatty acid in plant and animal tissue and the biosynthetic precursor of linoleic and linolenic acids (Christie, 2005), essential nutrients that are not synthesized by hymenopterans (Barbehenn, Reese, & Hagens, 1999; Canavoso, Jouni, Karnas, Pennington, & Wells, 2001; Dadd, 1973; Hagen, Dadd, & Reese, 1984). As the main constituent in insect hemolymph, oleic acid in the form of diolein is of particular nutritional importance for ant larvae (Fischer et al., 2008; Municio, Odriozola, & Pérez‐Albarsanz, 1975; Thompson, 1973). However, not all studies are in agreement that oleic acid is responsible for inducing carrying behavior in myrmecochorous systems. For example, O'Dowd and Hay (1980) found that elaiosomes of *Datura discolor* (Solanaceae) were conspicuously absent of oleic acid, whereas palmitic acid, stearic acid, linoleic acid, and linolenic acid were all present. We found that linoleic, hexadecanoic, stearic, and palmitoleic acids were also present in higher concentrations in the more attractive species of *Trillium*, so these fatty acids likely contribute to the overall attractiveness of seeds to ants.

Our prediction that elaiosome phytochemical profiles of the more attractive species of *Trillium* would be distinct from their less attractive congeners was supported. Although the partial RDA shows that all study species were independently clustered in multivariate space, the phytochemical profiles of the more attractive species, *T. catesbaei* and *T. cuneatum,* grouped out closer to one another than any of the less attractive species, suggesting greater similarity of their chemical profiles. The finding that sympatric endemic and widespread species have distinct elaiosome phytochemical profiles is noteworthy for two reasons. First, these species are closely related, minimizing differences in phytochemical composition that could be attributed to phylogeny. Second, when sympatric, these species may engage in interspecific competition for dispersal services; this suggests that the shared need to attract high‐quality ant dispersers may exert different strengths of selection on different species, or that other agents of selection differentially impact elaiosome phytochemistry in sympatric *Trillium* species. Other studies have produced similar findings for fatty acid chemistry in intraspecific populations of myrmecochores. Boulay et al. (2006) found that distant populations of *Helleborus foetidus* had significantly different fatty acid composition, which influenced preference in three major ant dispersers, and Boieiro et al. (2012) found that the content of oleic acid differed markedly among populations of *Euphorbia characias* in the Mediterranean. Environmental heterogeneity, lower rates of gene flow, or geographic variations in co‐adaptation between plant species and ant dispersers (i.e., less stabilizing selection exerted by ants) may explain the large disparity in the chemical profiles of the less attractive species. Narrowly endemic populations of myrmecochores may experience infrequent dispersal away from maternal patches, resulting in more heterogeneous chemical composition of their elaiosomes across populations due to low levels of gene flow. Indeed, the lower dispersal probabilities experienced by these endemic species (Miller & Kwit, 2018) supports this interpretation. In contrast, elaiosome phytochemistry of the widespread species might be more homogeneous due to higher levels of gene flow stemming from greater connectivity and higher rates of dispersal, potentially rendering their seeds more recognizable, and thus attractive, to ants.

Salicylate, xanthine, histidine, citrulline, and pantothenate were significantly negatively related to probability of seed dispersal according to follow‐up linear models to the partial RFRM. This finding is of interest, because very little research thus far has considered the impacts of repellent chemicals on myrmecochory. These metabolites, belonging to diverse classes including salicylic acids, purines, *α*‐amino acids, *L*‐*α*‐amino acids, and *β*‐amino acids, might function as deterrents to foraging ants making decisions about which items to pick up and carry back to the nest. In their study of European ant‐dispersed plants, Fischer et al. (2008) found citrulline to be present at very low concentrations in elaiosomes of all fifteen species considered. Low concentrations of citrulline in elaiosomes may therefore be favorable for ant‐mediated seed dispersal; alternatively, the presence of citrulline in elaiosomes may simply be a product of the Krebs‐Henseleit cycle, wherein arginine (a major constituent of seed protein), citrulline, and ornithine are metabolically interconverted (Hill‐Cottingham & Lloyd‐Jones, 1973). Davidson, Seidel, and Epstein (1990) discovered methyl‐6‐methyl salicylate, a compound closely related to salicylate, on the seeds of nine taxonomically unrelated ant garden epiphytes in western Amazonia; this compound was found to be mildly repellent to ants at high concentrations, but to stimulate ant excitement, seed handling, and seed carrying in lower concentrations in *Camponotus femoratus*. Our results corroborate these findings in that lower concentrations of salicylate were found in seeds that had a higher probability of being dispersed by ants. The potential roles played by the remaining compounds selected by random forest models is unknown, but could be the focus of further investigations.

The defense trade‐off hypothesis posited by Cipollini and Levey (1997), and further explored by Schaefer, Schmidt, and Winkler (2003), may explain the presence of phytochemicals that appear to reduce the attractiveness of trillium seeds. Plants must balance attracting dispersers and repelling the mortality agents that exposed fruits/seeds come into contact with prior to and following dispersal. Whereas some compounds present in elaiosomes might deter ant dispersers, this cost may be off‐set by better defenses against granivores or microbial pathogens. In myrmecochores, this could be of particular importance, given the likelihood that rodents will prey on seeds that are not removed by ants within a few hours of dehiscence (Heithaus, 1981). The importance of microbial pathogens has not been investigated thoroughly in myrmecochore systems, so deterrent compounds in elaiosomes might also play a role in defending the seed against fungal infections. The defense trade‐off hypothesis would imply that mortality selection agents (i.e., granivores, microbial pathogens) have had a greater impact on the less attractive, endemic species of *Trillium* considered here, which tend to contain higher concentrations of these deterrent compounds, whereas the widespread, attractive species might invest in nutrients and attractants resulting in their higher dispersal probabilities.

This study provides evidence that multiple physical and chemical diaspore traits influence how attractive the seeds of eastern North American species of *Trillium* are to ants, primarily of the keystone seed‐dispersing genus *Aphaenogaster*. Species within the *fulva‐rudis‐texana* complex, consisting of *A. fulva, A. rudis, A. texana,* and several other morphologically similar species including *A. picea* (Umphrey, 1996), are difficult to reliably distinguish from one another and may be characterized by polyphyly (DeMarco & Cognato, 2016). Our study system encompasses the ecotone between *A. picea* and *A. rudis* (Warren et al., 2016). Although both species are effective seed dispersers, they may be marked with differences in seed dispersal effectiveness that are not accounted for in this paper (see Warren, Bahn, & Bradford, 2011); as such, we acknowledge that some of the variability in seed dispersal probabilities across species of *Trillium* may be due to differences in the seed disperser assemblages at each study site. However, that pairs of the study species co‐occur within multiple sites and thus were exposed to identical dispersal assemblages during the in situ observations of seed dispersal likely minimizes these differences.

Based on our results, the trillium seeds that were most attractive to ants are characterized by larger diaspores overall (but not necessarily by larger elaiosome biomass or elaiosome‐seed mass ratios), and by elaiosomes with high concentrations of the *α*‐amino acid methylhistidine and the carbohydrate d‐glucarate, low concentrations of salicylate, xanthine, histidine, citrulline, and pantothenate, and high concentrations of free oleic, linoleic, hexadecenoic, stearic, palmitoleic, and total fatty acids. Many of these traits are likely correlated, and thus there is some redundancy in our multi‐dimensional description of an attractive myrmecochore seed. Our results regarding diaspore and elaiosome morphology and elaiosome fatty acids compliment the work of many previous studies, but we provide novel insights into the potential roles played by previously‐unknown components of the broader elaiosome phytochemical profile. Previous work in other *Trillium* species corroborates our conclusion that seed‐dispersing ants respond to a complex of characters, including morphological and chemical seed attributes (Gunther & Lanza, 1989; Lanza et al., 1992). Further advances in our understanding of seed dispersal effectiveness in myrmecochorous as well as other animal‐mediated seed dispersal systems will require a portrayal of dispersal unit chemical and physical traits, and their combined responses to selection pressures.

## AUTHOR CONTRIBUTIONS

C.N.M. and C.K. conceived of the ideas and designed methodology. C.N.M. collected materials from the field. C.N.M. and S.R.W. performed the experiments. C.N.M. and S.R.W. analyzed the data. C.N.M., C.K., and S.R.W. wrote the manuscript.

## Data Availability

Data are available from the Dryad Digital Repository: https://doi.org/10.5061/dryad.hhmgqnkcz

## References

[ece36101-bib-0001] Barbehenn, R. V. , Reese, J. C. , & Hagens, K. S. (1999). The food of insects In HuVaker C. B., & Gutierrez A. P. (Eds.), Ecological entomology, 2nd ed. (pp. 83–121). New York: Wiley. ISBN 047124483X, 9780471244837.

[ece36101-bib-0003] Biau, G. (2012). Analysis of a random forests model. Journal of Machine Learning Research, 13, 1063–1095.

[ece36101-bib-0004] Bielby, J. , Cardillo, M. , Cooper, N. , & Purvis, A. (2010). Modelling extinction risk in multispecies data sets: Phylogenetically independent contrasts versus decision trees. Biodiversity and Conservation, 19, 113–127. 10.1007/s10531-009-9709-0

[ece36101-bib-0005] Bluthgen, N. , & Fiedler, K. (2004). Competition for composition: Lessons from nectar‐feeding ant communities. Ecology, 85, 1479–1485. 10.1890/03-0430

[ece36101-bib-0006] Bluthgen, N. , Gottsberger, G. , & Fiedler, K. (2004). Sugar and amino acid composition of ant‐attended nectar and honeydew sources from an Australian rainforest. Austral Ecology, 29, 418–429. 10.1111/j.1442-9993.2004.01380.x

[ece36101-bib-0007] Boieiro, M. , Espadaler, X. , Gómez, C. , & Eustaquio, A. (2012). Spatial variation in the fatty acid composition of elaiosomes in an ant‐dispersed plant: Differences within and between individuals and populations. Flora‐Morphology, Distribution, Functional Ecology of Plants, 207(7), 497–502. 10.1016/j.flora.2012.06.007

[ece36101-bib-0008] Boulay, R. , Coll‐Toledano, J. , & Cerdá, X. (2006). Geographic variations in *Helleborus foetidus* elaiosome lipid composition: Implications for dispersal by ants. Chemoecology, 16, 1–7. 10.1007/s00049-005-0322-8

[ece36101-bib-0009] Boulay, R. , Coll‐Toledano, J. , Manzaneda, A. J. , & Cerdá, X. (2007). Geographic variations in seed dispersal by ants: Are plant and seed traits decisive? Naturwissenschaften, 94(3), 242–246. 10.1007/s00114-006-0185-z 17119907

[ece36101-bib-0010] Bresinsky, A. (1963). Bau, Entwicklungsgeschichte und Inhaltsstoffe der‐Elaiosomen.

[ece36101-bib-0011] Brew, C. R. , O'Dowd, D. J. , & Rae, I. A. (1989). Seed dispersal by ants: Behaviour‐releasing compounds in elaiosomes. Oecologia, 80, 490–497. 10.1007/BF00380071 28312833

[ece36101-bib-0012] Buono, R. A. , De Oliveira, A. B. , & Sousa Paiva, E. A. (2008). Anatomy, ultrastructure and chemical composition of food bodies of *Hovenia dulcis* (Rhamnaceae). Annals of Botany, 101, 1341–1348. 10.1093/aob/mcn052 18413656 PMC2710260

[ece36101-bib-0013] Canavoso, L. E. , Jouni, Z. E. , Karnas, K. J. , Pennington, J. E. , & Wells, M. A. (2001). Fat metabolism in insects. Annual Review of Nutrition, 21, 23–46. 10.1146/annurev.nutr.21.1.23 11375428

[ece36101-bib-0014] Caut, S. , Jowers, M. J. , Cerdá, X. , & Boulay, R. R. (2013). Questioning the mutual benefits of myrmecochory: A stable isotope‐based experimental approach. Ecological Entomology, 38(4), 390–399. 10.1111/een.12028

[ece36101-bib-0015] Christie, W. W. (2005). The lipid library. http://www.lipidlibrary.co.uk.

[ece36101-bib-0016] Cipollini, M. L. , & Levey, D. J. (1997). Secondary metabolites of fleshy vertebrate‐dispersed fruits: adaptive hypotheses and implications for seed dispersal. The American Naturalist, 150(3), 346–372. 10.1086/286069 18811294

[ece36101-bib-0017] Clasquin, M. F. , Melamud, E. , & Rabinowitz, J. D. (2012). LC‐MS data processing with MAVEN: A metabolomic analysis and visualization engine. Current Protocols in Bioinformatics, 37(1), 14–11. 10.1002/0471250953.bi1411s37 PMC4055029 22389014

[ece36101-bib-0018] Dadd, R. H. (1973). Insect nutrition: Current developments and metabolic implications. Annual Review of Entomology, 18, 381–429. 10.1146/annurev.en.18.010173.002121 4218469

[ece36101-bib-0019] Davidson, D. W. , Foster, R. B. , Snelling, R. R. , & Lozada, P. W. (1991). Variable composition of some tropical ant–plant symbioses In Price P. W., Lewinsohn T. M., Fernandes G. W., & Benson W. W. (Eds.), Plant‐animal interactions: Evolutionary ecology in tropical and temperate regions (pp. 145–162). New York: USA, Wiley.

[ece36101-bib-0020] Davidson, D. W. , Seidel, J. L. , & Epstein, W. W. (1990). Neotropical ant gardens II. Bioassays of seed compounds. Journal of Chemical Ecology, 16(10), 2993–3013. 24263271 10.1007/BF00979490

[ece36101-bib-0021] DeMarco, B. B. , & Cognato, A. I. (2016). A multiple‐gene phylogeny reveals polyphyly among eastern North American *Aphaenogaster* species (Hymenoptera: Formicidae). Zoologica Scripta, 45(5), 512–520.

[ece36101-bib-0022] Djoumbou Feunang, Y. , Eisner, R. , Knox, C. , Chepelev, L. , Hastings, J. , Owen, G. , … Wishart, D. S. (2016). ClassyFire: Automated chemical classification with a comprehensive, computable taxonomy. Journal of Cheminformatics, 8(1), 61 10.1186/s13321-016-0174-y 27867422 PMC5096306

[ece36101-bib-0023] Edwards, W. , Dunlop, M. , & Rodgerson, L. (2006). The evolution of rewards: Seed dispersal, seed size and elaiosome size. Journal of Ecology, 94, 687–694. 10.1111/j.1365-2745.2006.01116.x

[ece36101-bib-0024] Fischer, R. C. , Ölzant, S. M. , Wanek, W. , & Mayer, V. (2005). The fate of *Corydalis cava* elaiosomes within an ant colony of *Myrmica rubra*: Elaiosomes are preferentially fed to larvae. Insectes Sociaux, 52(1), 55–62. 10.1007/s00040-004-0773-x

[ece36101-bib-0025] Fischer, R. C. , Richter, A. , Hadacek, F. , & Mayer, V. (2008). Chemical differences between seeds and elaiosomes indicate an adaptation to nutritional needs of ants. Oecologia, 155, 539–547. 10.1007/s00442-007-0931-8 18095003

[ece36101-bib-0026] Fischer, R. C. , Richter, A. , Wanek, W. , & Mayer, V. (2002). Plants feed ants: Food bodies of myrmecophytic *Piper* and their significance for the interaction with *Pheidole bicornis* ants. Oecologia, 133, 186–192. 10.1007/s00442-002-1000-y 28547305

[ece36101-bib-0027] Fokuhl, G. , Heinze, J. , & Poschlod, P. (2007). Colony growth in *Myrmica rubra* with supplementation of myrmecochorous seeds. Ecological Research, 22(5), 845–847. 10.1007/s11284-006-0331-2

[ece36101-bib-0028] Freeman, J. D. (1975). Revision of *Trillium* subgenus Phyllantherum (Liliaceae). Brittonia, 27(1), 1–62. 10.2307/2805646

[ece36101-bib-0029] Fricke, E. C. , Haak, D. C. , Levey, D. J. , & Tewksbury, J. J. (2016). Gut passage and secondary metabolites alter the source of post‐dispersal predation for bird‐dispersed chili seeds. Oecologia, 181(3), 905–910. 10.1007/s00442-016-3612-7 27016078

[ece36101-bib-0030] Gaddy, L. (1986). Twelve new ant‐dispersed species in the Southern Appalachians. Bulletin of the Torrey Botanical Club, 113, 247–251.

[ece36101-bib-0031] Gammans, N. , Bullock, J. M. , Gibbons, H. , & Schönrogge, K. (2006). Reaction of mutualistic and granivorous ants to *Ulex* elaiosome chemicals. Journal of Chemical Ecology, 32(9), 1935–1947. 10.1007/s10886-006-9119-7 16902826

[ece36101-bib-0032] Gonzalez‐Teuber, M. , & Heil, M. (2009). The role of extrafloral nectar amino acids for the preferences of facultative and obligate ant mutualists. Journal of Chemical Ecology, 35, 459–468. 10.1007/s10886-009-9618-4 19370376

[ece36101-bib-0033] Gorb, S. N. , & Gorb, E. V. (1995). Removal rates of seeds of five myrmecochorous plants by the ant *Formica polyctena* (Hymenoptera: Formicidae). Oikos, 367–374. 10.2307/3545960

[ece36101-bib-0034] Gordon, S. C. , Meadley‐Dunphy, S. A. , Prior, K. M. , & Frederickson, M. E. (2019). Asynchrony between ant seed dispersal activity and fruit dehiscence of myrmecochorous plants. American Journal of Botany, 106(1), 71–80. 10.1002/ajb2.1214 30644530

[ece36101-bib-0035] Gove, A. D. , Majer, J. D. , & Dunn, R. R. (2007). A keystone ant species promotes seed dispersal in a “diffuse” mutualism. Oecologia, 153(3), 687–697. 10.1007/s00442-007-0756-5 17534665

[ece36101-bib-0036] Gunther, R. W. , & Lanza, J. (1989). Variation in attractiveness of *Trillium* diaspores to a seed‐dispersing ant. American Midland Naturalist, 321–328. 10.2307/2425919

[ece36101-bib-0037] Hagen, K. S. , Dadd, R. H. , & Reese, J. (1984). The food of insects In HuVaker C. B., & Rabb R. L. (Eds.), Ecological entomology (pp. 79–111). New York: Wiley.

[ece36101-bib-0038] Heil, M. (2011). Nectar: Generation, regulation, and ecological functions. Trends in Plant Science, 16, 191–200. 10.1016/j.tplants.2011.01.003 21345715

[ece36101-bib-0039] Heil, M. , Baumann, B. , Kruger, R. , & Linsenmair, K. E. (2004). Main nutrient compounds in food bodies of Mexican *Acacia* ant‐plants. Chemoecology, 14, 45–52. 10.1007/s00049-003-0257-x

[ece36101-bib-0040] Heil, M. , Fiala, B. , Kaiser, W. , & Linsenmair, K. E. (1998). Chemical contents of Macaranga food bodies: Adaptations to their role in ant attraction and nutrition. Functional Ecology, 12, 117–122. 10.1046/j.1365-2435.1998.00158.x

[ece36101-bib-0041] Heithaus, E. R. (1981). Seed predation by rodents on three ant‐dispersed plants. Ecology, 62(1), 136–145. 10.2307/1936677

[ece36101-bib-0042] Hill‐Cottingham, D. G. , & Lloyd‐Jones, C. P. (1973). Metabolism of carbon‐14 labelled arginine, citrulline and ornithine in intact apple stems. Physiologia Plantarum, 29(1), 125–128. 10.1111/j.1399-3054.1973.tb04822.x

[ece36101-bib-0043] Hothorn, T. , Bretz, F. , & Westfall, P. (2008). Simultaneous inference in general parametric models. Biometrical Journal, 50(3), 346–363. 10.1002/bimj.200810425 18481363

[ece36101-bib-0045] Hughes, L. , & Westoby, M. (1992). Effect of diaspore characteristics on removal of seeds adapted for dispersal by ants. Ecology, 73, 1300–1312. 10.2307/1940677

[ece36101-bib-0046] Hughes, L. , Westoby, M. T. , & Jurado, E. (1994). Convergence of elaiosomes and insect prey: Evidence from ant foraging behaviour and fatty acid composition. Functional Ecology, 358–365. 10.2307/2389829

[ece36101-bib-0048] Keene, O. N. (1995). The log transformation is special. Statistics in Medicine, 14(8), 811–819. 10.1002/sim.4780140810 7644861

[ece36101-bib-0049] Keselman, H. J. , Algina, J. , Kowalchuk, R. K. , & Wolfinger, R. D. (1999). The analysis of repeated measurements: A comparison of mixed‐model Satterthwaite F tests and a nonpooled adjusted degrees of freedom multivariate test. Communications in Statistics‐Theory and Methods, 28(12), 2967–2999. 10.1080/03610929908832460

[ece36101-bib-0050] Kursa, M. B. , & Rudnicki, W. R. (2010). Feature selection with the Boruta package. Journal of Statistical Software, 36(11), 1–13.

[ece36101-bib-0051] Kusmenoglu, S. , Rockwood, L. L. , & Gretz, M. R. (1989). Fatty acids and diacylglycerols from elaiosomes of some ant‐dispersed seeds. Phytochemistry, 28(10), 2601–2602. 10.1016/S0031-9422(00)98048-8

[ece36101-bib-0052] Kuznetsova, A. , Brockhoff, P. B. , & Christensen, R. B. (2017). lmerTest Package: Tests in linear mixed effects models. Journal of Statistical Software, 82(13), 1–26. 10.18637/jss.v082.i13

[ece36101-bib-0053] Lanza, J. , Schmitt, M. A. , & Awad, A. B. (1992). Comparative chemistry of elaiosomes of three species of *Trillium* . Journal of Chemical Ecology, 18(2), 209–221. 10.1007/BF00993754 24254910

[ece36101-bib-0054] Leal, L. , Lima Neto, M. , de Oliveira, A. , Andersen, A. , & Leal, I. (2014). Myrmecochores can target high‐quality disperser ants: Variation in elaiosome traits and ant preferences for myrmecochorous Euphorbiaceae in Brazilian Caatinga. Oecologia, 174, 493–500. 10.1007/s00442-013-2789-2 24085639

[ece36101-bib-0055] Legendre, P. , & Legendre, L. (1998). Numerical Ecology, 2nd ed. Amsterdam: Elsevier. ISBN 978‐0444892508.

[ece36101-bib-0056] Levine, N. , Ben‐Zevi, G. , Seifan, M. , & Giladi, I. (2019). Investment in reward by ant‐dispersed plants consistently selects for better partners along a geographic gradient. AoB PLANTS, 11(3), 1–13. 10.1093/aobpla/plz027 PMC6534284 31139335

[ece36101-bib-0057] Liaw, A. , & Wiener, M. (2002). Classification and regression by randomForest. R News, 2(3), 18–22.

[ece36101-bib-0058] Lisci, M. , Bianchini, M. , & Pacini, E. (1996). Structure and function of the elaiosome in some angiosperm species. Flora, 191(2), 131–141. 10.1016/S0367-2530(17)30704-1

[ece36101-bib-0059] Manzaneda, A. J. , & Rey, P. J. (2009). Assessing ecological specialization of an ant–seed dispersal mutualism through a wide geographic range. Ecology, 90(11), 3009–3022. 10.1890/08-2274.1 19967857

[ece36101-bib-0060] Manzaneda, A. J. , Rey, P. J. , & Alcántara, J. M. (2009). Conflicting selection on diaspore traits limits the evolutionary potential of seed dispersal by ants. Journal of Evolutionary Biology, 22(7), 1407–1417. 10.1111/j.1420-9101.2009.01752.x 19460082

[ece36101-bib-0061] Mark, S. , & Olesen, J. (1995). Importance of elaiosome size to removal of ant‐dispersed seeds. Oecologia, 107, 95–101. 10.1007/BF00582239 28307196

[ece36101-bib-0062] Marshall, D. L. , Beattie, A. J. , & Bollenbacher, W. E. (1979). Evidence for diglycerides as attractants in an ant‐seed interaction. Journal of Chemical Ecology, 5(3), 335–344. 10.1007/BF00987919

[ece36101-bib-0063] Mayer, V. E. , Frederickson, M. E. , McKey, D. , & Blatrix, R. (2014). Current issues in the evolutionary ecology of ant–plant symbioses. New Phytologist, 202(3), 749–764. 10.1111/nph.12690 24444030

[ece36101-bib-0064] Miller, C. N. , & Kwit, C. (2018). Overall seed dispersal effectiveness is lower in endemic *Trillium* species than in their widespread congeners. American Journal of Botany, 105(11), 1–11. 10.1002/ajb2.1188 30383896

[ece36101-bib-0065] Morrone, O. , Vega, A. S. , & Maier, M. (2000). Elaiosomes in *Urochloa paucispicata* (Poaceae: Panicoideae: Paniceae): Anatomy and chemical composition. Flora, 195(4), 303–310. 10.1016/S0367-2530(17)30989-1

[ece36101-bib-0066] Municio, A. A. , Odriozola, J. M. , & Pérez‐Albarsanz, A. (1975). Biochemistry of development in insects. European Journal of Biochemistry, 60, 123–128. 1204633 10.1111/j.1432-1033.1975.tb20983.x

[ece36101-bib-0068] Nelson, A. S. , Carvajal Acosta, N. C. , & Mooney, K. A. (2019). Plant chemical mediation of ant behavior. Current Opinion in Insect Science, 32, 98–103. 10.1016/j.cois.2018.12.003 31113639

[ece36101-bib-0069] Ness, J. H. , Bronstein, J. L. , Andersen, A. N. , & Holland, J. N. (2004). Ant body size predicts dispersal distance of ant‐adapted seeds: Implications of small‐ant invasions. Ecology, 85, 1244–1250. 10.1890/03-0364

[ece36101-bib-0070] Ness, J. H. , Morin, D. , & Giladi, I. (2009). Uncommon specialization in a mutualism between a temperate herbaceous plant guild and an ant: Are *Aphaenogaster* ants keystone mutualists? Oikos, 118, 1793–1804.

[ece36101-bib-0071] O'Dowd, D. J. , & Hay, M. E. (1980). Mutualism between harvester ants and a desert ephemeral: Seed escape from rodents. Ecology, 61(3), 531–540.

[ece36101-bib-0072] Ohara, M. (1989). Life history evolution in the genus *Trillium* . Plant Species Biology, 4, 1–28. 10.1111/j.1442-1984.1989.tb00044.x

[ece36101-bib-0073] Oksanen, J. , Blanchet, F. G. , Kindt, R. , Legendre, P. , Minchin, P. R. , O'hara, R. B. , … Wagner, H. (2017). Vegan: Community ecology package. R Package Version, 2(3–0), 2015.

[ece36101-bib-0074] Osborne, J. (2002). Notes on the use of data transformations. Practical Assessment, Research, and Evaluation, 8(1), 6.

[ece36101-bib-0075] Paiva, E. A. S. , Buono, R. A. , & Lombardi, J. A. (2009). Food bodies in *Cissus verticillata* (Vitaceae): Ontogenesis, structure and functional aspects. Annals of Botany, 103, 517–524. 10.1093/aob/mcn237 19049986 PMC2707332

[ece36101-bib-0077] Pfeiffer, M. , Huttenlocher, H. , & Ayasse, M. (2010). Myrmecochorous plants use chemical mimicry to cheat seed‐dispersing ants. Functional Ecology, 24, 545–555. 10.1111/j.1365-2435.2009.01661.x

[ece36101-bib-0078] Qiu, H. L. , Lu, L. H. , Shi, Q. X. , Tu, C. C. , Lin, T. , & He, Y. R. (2015). Differential necrophoric behaviour of the ant *Solenopsis invicta* towards fungal‐infected corpses of workers and pupae. Bulletin of Entomological Research, 105(5), 607–614. 26082426 10.1017/S0007485315000528

[ece36101-bib-0079] R Core Team . (2017). R: A language and environment for statistical computing. Vienna, Austria: R Foundation for Statistical Computing.

[ece36101-bib-0081] Reifenrath, K. , Becker, C. , & Poethke, H. J. (2012). Diaspore trait preferences of dispersing ants. Journal of Chemical Ecology, 38, 1093–1104. 10.1007/s10886-012-0174-y 22903746

[ece36101-bib-0082] Rickson, F. (1976). Ultrastructural differentiation of Mullerian body glycogen plastid of *Cecropia peltata* L. American Journal of Botany, 63, 1272–1279.

[ece36101-bib-0084] Schaefer, H. M. , Schmidt, V. , & Winkler, H. (2003). Testing the defence trade‐off hypothesis: How contents of nutrients and secondary compounds affect fruit removal. Oikos, 102(2), 318–328. 10.1034/j.1600-0706.2003.11796.x

[ece36101-bib-0085] Schupp, E. W. (1993). Quantity, quality and the effectiveness of seed dispersal by animals. Vegetatio, 107(1), 15–29.

[ece36101-bib-0086] Schupp, E. , Jordano, P. , & Gómez, J. (2010). Seed dispersal effectiveness revisited: A conceptual review. New Phytologist, 188, 333–353. 10.1111/j.1469-8137.2010.03402.x 20673283

[ece36101-bib-0087] Shenoy, M. , Radhika, V. , Satish, S. , & Borges, R. M. (2012). Composition of extrafloral nectar influences interactions between the myrmecophyte *Humboldtia brunonis* and its ant associates. Journal of Chemical Ecology, 38, 88–99. 10.1007/s10886-011-0052-z 22234428

[ece36101-bib-0088] Skidmore, B. A. , & Heithaus, E. R. (1988). Lipid cues for seed‐carrying by ants in *Hepatica americana* . Journal of Chemical Ecology, 14(12), 2185–2196. 10.1007/BF01014024 24277238

[ece36101-bib-0089] Soukup, V. G. , & Holman, R. T. (1987). Fatty acids of seeds of North American pedicellate *Trillium* species. Phytochemistry, 26(4), 1015–1018.

[ece36101-bib-0090] Takahashi, S. , & Itino, T. (2015). Larger seeds are dispersed farther: The long distance seed disperser ant *Aphaenogaster famelica* prefers larger seeds. Sociobiology, 59, 1401–1411.

[ece36101-bib-0091] Tewksbury, J. J. , & Nabhan, G. P. (2001). Seed dispersal: Directed deterrence by capsaicin in chillies. Nature, 412(6845), 403. 11473305 10.1038/35086653

[ece36101-bib-0092] Tewksbury, J. J. , Reagan, K. M. , Machnicki, N. J. , Carlo, T. A. , Haak, D. C. , Peñaloza, A. L. C. , & Levey, D. J. (2008). Evolutionary ecology of pungency in wild chilies. Proceedings of the National Academy of Sciences, 105(33), 11808–11811. 10.1073/pnas.0802691105 PMC2575311 18695236

[ece36101-bib-0093] Thompson, S. N. (1973). A review and comparative characterization of the fatty acid composition of seven insect orders. Comparative Biochemistry and Physiology, 45B, 467–482.

[ece36101-bib-0094] Turner, K. , & Frederickson, M. (2013). Signals can trump rewards in attracting seed‐dispersing ants. PLoS ONE, 8, 1–9. 10.1371/journal.pone.0071871 PMC3742508 23967257

[ece36101-bib-0095] Umphrey, G. J. (1996). Morphometric discrimination among sibling species in the *fulva–rudis–texana* complex of the ant genus *Aphaenogaster* (Hymenoptera: Formicidae). Canadian Journal of Zoology, 74(3), 528–559.

[ece36101-bib-0096] Van Der Pijl, L. (1982). Principles of dispersal in higher plants. New York: Springer‐Verlag.

[ece36101-bib-0097] Warren, R. J. , Bahn, V. , & Bradford, M. A. (2011). Temperature cues phenological synchrony in ant‐mediated seed dispersal. Global Change Biology, 17(7), 2444–2454. 10.1111/j.1365-2486.2010.02386.x

[ece36101-bib-0098] Warren, R. J. , Chick, L. D. , DeMarco, B. , McMillan, A. , De Stefano, V. , Gibson, R. , & Pinzone, P. (2016). Climate‐driven range shift prompts species replacement. Insectes Sociaux, 63(4), 593–601. 10.1007/s00040-016-0504-0

[ece36101-bib-0099] Warren, R. J. , Giladi, I. , & Bradford, M. A. (2014). Competition as a mechanism structuring mutualisms. Journal of Ecology, 102, 486–495. 10.1111/1365-2745.12203

[ece36101-bib-0100] Webber, B. L. , Abaloz, B. A. , & Woodrow, I. E. (2007). Myrmecophilic food body production in the understorey tree, *Ryparosa kurrangii* (Achariaceae), a rare Australian rainforest taxon. New Phytologist, 173, 250–263. 17204073 10.1111/j.1469-8137.2006.01916.x

[ece36101-bib-0101] Wenig, P. , & Odermatt, J. (2010). OpenChrom: A cross‐platform open source software for the mass spectrometric analysis of chromatographic data. BMC Bioinformatics, 11(1), 405 10.1186/1471-2105-11-405 20673335 PMC2920884

[ece36101-bib-0102] Wu, J. , Peng, L. , Dong, S. , Xia, X. , & Zhao, L. (2019). Transcriptome analysis of *Chelidonium majus* elaiosomes and seeds provide insights into fatty acid biosynthesis. PeerJ, 7, e6871 10.7717/peerj.6871 31110927 PMC6501766

[ece36101-bib-0103] Zelikova, T. J. , Sanders, N. J. , & Dunn, R. (2011). The mixed effects of experimental ant removal on seedling distribution, belowground invertebrates, and soil nutrients. Ecosphere, 2, 1–14. 10.1890/ES11-00073.1

