# Supplementary material for: Effects of seed morphology and elaiosome chemical composition on attractiveness of five Trillium species to seed‐dispersing ants
Source: Ecol Evol. 2020 Feb 27;10(6):2860–73. doi: 10.1002/ece3.6101 (PMC7083703; doi:10.1002/ece3.6101)
Supplement: Supplementary file 1 [file ECE3-10-2860-s001.docx]

**Supplemental Materials**

**Manuscript:** Effects of seed morphology and elaiosome chemical composition on attractiveness of five *Trillium* species to seed-dispersing ants

**Authors:** Miller, C. N., Whitehead, S. R., & Kwit, C.

**Methods**

*Liquid chromatography-mass spectrometry*

Using sterile techniques, we removed elaiosomes from frozen diaspores of each species using a straight razor. We recorded the fresh mass of single elaiosomes (g) and placed each elaiosome in a 2.0 mL centrifuge tube. Sample contents were suspended in 1.3 mL of extraction solvent (40 : 40 : 20 HPLC grade methanol, acetonitrile, water with 0.1% formic acid), and were kept at 4°C. Extraction proceeded for 20 min at -20°C before samples were centrifuged for 5 min (16.1 relative centrifugal force [rcf]) at 4°C. Supernatants were transferred to new vials. The remaining elaiosome contents were resuspended in 200 μL of cold (4°C) extraction solvent. The extraction again proceeded for 20 min at -20°C before being centrifuged for 5 min (16.1 rcf) at 4°C. Once more, we transferred supernatants to the vials, and added another 200 μL of extraction solvent to the pelleted elaiosomes for a final wash by repeating the extraction once more. The vials containing all of the combined extraction supernatants were then placed in a nitrogen drying apparatus until all the extraction solvent had been evaporated. We resuspended the residue in 300 μL of sterile water to isolate the polar fraction of phytochemicals and transferred this to 300 μL autosampler vials. Samples were immediately placed in a 4°C autosampler for LC-MS analysis.

  A 10 μL injection of each sample was separated through a Synergi 2.5 micron Hydro-RP 100 Å, 100 mm × 2.00 mm LC column (Phenomenex, Torrance, CA, USA) maintained at 25°C. The mass spectrometer and chromatographic separation were performed similar to the method outlined in Lu et al., 2010. The eluent was introduced into the mass spectrometer via an electrospray ionization source in negative mode before entering an Exactive Plus orbitrap mass spectrometer (Thermo Scientific, Waltham, MA, USA) through a 0.1-mm internal diameter fused silica capillary tube. Samples were run with a spray voltage of 3 kV, a nitrogen sheath gas flow rate of 10 units, a capillary temperature set at 320°C, and an AGC target set to 3e6. Samples were analyzed in full scan mode with a resolution of 140,000 and a scan window of 85 to 800 m/z for from 0 to 9 min and 110 to 1000 m/z from 9 to 25 min. Solvent A consisted of 97:3 HPLC grade water : methanol, 10 mM tributylamine, and 15 mM acetic acid. Solvent B was HPLC grade methanol. The mobile phase gradient from 0 to 5 min was 0% B, from 5 to 13 min was 20% B, from 13 to 15.5 min was 55% B, from 15.5 to 19 min is 95% B, and from 19 to 25 min was 0% B while maintaining a constant flow rate of of 200 μL/min.

Raw files obtained from Xcalibur MS software (Thermo Electron Corp., Waltham, MA) were converted into the mzML format using ProteoWizard (Chambers et al., 2012). The converted files were imported into MAVEN (Metabolomic Analysis and Visualization Engine for LC–MS Data), a software package (Clasquin et al. 2012). Peaks for the known metabolites were picked in MAVEN, which automatically performs non-linear retention time correction and calculates peak areas across samples, using a preliminary mass error of ± 20 ppm and retention time window of 5 min. The University of Tennessee, Knoxville Biological and Small Molecule Mass Spectrometry Core (BSMMSC), through which this analysis took place, has replicated and expanded the method of Rabinowitz and coworkers (Lu et al., 2010). Final metabolite annotations were made using a library of 263 retention time-accurate m/z pairs taken from MS1 spectra. The annotation parameters have been verified previously with pure standards in the course of establishing the method. For a metabolite to be annotated as a known compound, the eluted peak had to be found within 2 min of the expected retention time, and the metabolite mass had to be within ± 5 ppm of the expected value. Metabolite identities were confirmed using the MAVEN software package (Clasquin et al., 2012), and peak areas for each compound were integrated using the Quan Browser function of the Xcalibur MS Software.

*Gas-chromatography mass spectrometry*

We removed elaiosomes from frozen seeds as described above and placed them in 15-mL glass scintillation vials. Twelve samples were generated, representing the five study species, each from at least two field sites. Each sample contained a minimum of 30 mg of elaiosomes from one individual plant. The number of elaiosomes used for each sample depended on the species but ranged from 14 to 35. To each sample, we added 2.0 mL of the extraction solvent (2 : 1 dichloromethane : methanol). Samples were centrifuged for 30 m (170 rpm), and supernatants were siphoned off and transferred to new glass scintillation vials. We repeated this treatment three times, until 6 mL of supernatants were generated for each sample. The vials containing all of the combined extraction supernatants were placed in a nitrogen drying apparatus until all the extraction solvent had been evaporated. The dry residue in each vial was suspended in 1 mL isooctane : ethyl acetate (10 : 1) and was then applied to an alumina column (4-cm Pasteur pipette filled with flash alumina pre-equilibrated with isooctane : ethyl acetate 10 : 1), as in Boulay et al. (2006). The column was eluted with 4 ml isooctane : ethyl acetate (10 : 1) to yield triglyceride fractions for each sample. Elution of the diglyceride fraction was realized with 5 mL of isooctane : ethyl acetate (3 : 1). The free fatty acids were captured after elution with 6 mL of isooctane : ethyl acetate : acetic acid (75 : 25 : 2). Fractions were collected in standard glass test tubes.

Resulting fractions of triglycerides, diglycerides, and free fatty acids were dried under nitrogen for 3 h. Residue was dissolved in 1 mL of the extraction solvent, and then 400 μL of this volume was transferred to a 2.0 mL vial containing 20 μg of octadecane in methanol as an internal standard. Solutions were dried again under nitrogen and subsequent steps taken to convert fatty acids to their methyl esters for GC-MS analysis. Transesterification for tri- and diglyceride fractions was accomplished by adding 100 µL of methanolic KOH (0.5 M) to the dry triglyceride and diglyceride fractions. After 30 m, the reaction was stopped with 100 µL of HCl (1 N). Then 20 µL of hexane was added to the mixture. Methylation of the free fatty acid fraction was carried out by treating the solution with 200 µL of toluene, 1.5 mL of methanol, and 300 µL of an 8% (w/v) solution of HCL in methanol/water (85 : 15, w/v), sequentially, to the fraction, as in Ichihara and Fukubayashi (2009). The fraction was then heated to 100 C for 1 h. All the obtained fractions were dried one final time and dissolved in 100 μL of isooctane, of which 1 µL was injected into an Agilent 7820 gas chromatograph coupled with a 5977 mass spectrometer set to 70eV electron ionization and equipped with an HP5-MS column (30 m x 0.25 mm i.d., 0.25 μm film thickness; Agilent Technologies, Santa Clara, CA, USA). Ultra-pure helium was used as a carrier gas at a flow rate of 1 mL min -1, a split flow ratio of 100:1, and a front inlet temperature of 275 °C. The following oven conditions were employed: initial temperature 120 °C, hold time 2 min; ramp 1: 15°C min^-1^ to 250 °C, hold time 0 min; ramp 2: 5 °C min^-1^ to 300°C, hold time 10 min; total run time of 30.67 min. Data were recorded as TIC chromatograms.

*Comparisons of total fatty acids, and fatty acids in three forms (free, di-, and triglycerides)*

To enable linear comparisons of concentrations of fatty acids present in total, free form, and di- and triglycerides, we built simple linear models with the averaged probability of seed dispersal for each species as the predictor and percent fresh weight concentrations of total fatty acids, summed free fatty acids, and summed fatty acids in diglycerides and triglycerides as the respective responses. We generated best fit lines for each regression to illustrate the direction of trends.

**Results**

*Full RFCM and RFRM*

The full (known + unknown) phytochemical composition of elaiosomes were distinguishable among species of *Trillium* based on the RFCM. The out-of-bag error rate was 0.73, meaning the model only had 27% sample classification accuracy. Boruta variable selection identified 18 of the possible 7,552 variables as important classifiers. A MANOVA showed a significant overall difference among species for these 18 compounds (F_36,80_=8.28, *P*<0.001). Follow-up ANOVAs revealed that 15 of the 19 compounds were significantly different among the five species (Supplemental Materials Table 2). The full phytochemical composition of elaiosomes was distinguishable among averaged seed dispersal probabilities based on the RFRM, although the model only explained 0.77% of the variation in the data. The full RFRM identified 14 of the possible 7,552 variables as important in distinguishing among averaged dispersal probabilities. Follow-up linear models revealed that 10 of the 14 variables were significantly different among the species. Of the 14 compounds identified by the full RFRM, 5 compounds were also identified by the full RFCM (methylhistidine, creatinol O-phosphate, citrulline, d-glucarate, and indole 3-carboxylate; Supplemental Materials Table 2).

*Comparisons of total fatty acids, and fatty acids in three forms (free, di-, and triglycerides)*

Concentrations of oleic, linoleic, hexadecenoic, stearic, and total fatty acids differed significantly among the three fatty acid forms (*X^2^*(2)=15.43, *P*<0.001; *X^2^*(2)=29.34, *P*<0.001; *X^2^*(2)=30.93, *P*<0.001; *X^2^*(2)=211.57, *P*<0.001; *X^2^*(2)=19.51, *P*<0.001, respectively). Post hoc tests revealed that concentrations of oleic, linoleic, hexadecenoic, and total fatty acids were significantly greater in triglyceride forms than in diglyceride forms, with free fatty acid forms intermediate; concentrations of stearic acid were significantly higher in free form than either di- or triglycerides (Supplemental Materials Table 1; Supplemental Materials Fig. 3). The concentration of palmitoleic acid was not significantly different among the three forms (*X^2^*(2)=5.26, *P*=0.07). Concentrations of summed total fatty acids and summed free fatty acids were significantly positively related to seed dispersal probability (t=2.9, *P*=0.05; t=2.8, *P*=0.02, respectively). Concentrations of summed fatty acids in diglycerides and triglycerides were not significantly related to seed dispersal probability, although both trends are positive (Supplemental Materials Fig. 4).

Concentrations of all fatty acids except palmitoleic acid were significantly different in free, diglyceride, and triglyceride forms, and post hoc tests revealed that concentrations tended to be lowest in diglycerides and highest in free fatty acids and triglycerides. Linear trends showed that only summed total fatty acids and summed free fatty acids were significantly, positively related to the probability of seed dispersal. It is interesting that only summed free fatty acids, not summed triglycerides, were significantly related to dispersal probability, given that specific fatty acids were present in high concentrations in triglycerides. Of the three forms, free fatty acids may be most important to ants in this study system, corroborating the findings of Boulay, et al. (2006) and Pfeiffer, et al. (2010), but disagreeing with Marshall, et al. (1979) and Gammans, et al. (2006).

**Table 1.** Tukey post hoc comparisons following linear mixed-effects models for diaspore morphological metrics and elaiosome fatty acids that were found to be significantly different among the species of *Trillium.* Significant *P*-values are in bold.

| **Post Hoc comparisons** | **Linear Hypothesis** |  |  | **Est.** | **S.E.** | **Z-value** | **P-value** |
| --- | --- | --- | --- | --- | --- | --- | --- |
| **Diaspore mass** | cuneatum | - | catesbaei | -0.0071225 | 0.001843 | -3.865 | **<0.001** |
|  | decumbens | - | catesbaei | -0.0116465 | 0.0020864 | -5.582 | **<0.001** |
|  | discolor | - | catesbaei | -0.0014638 | 0.0009875 | -1.482 | 0.69 |
|  | lancifolium | - | catesbaei | -0.0054543 | 0.0093268 | -0.585 | 1 |
|  | decumbens | - | cuneatum | -0.004524 | 0.0010062 | -4.496 | **<0.001** |
|  | discolor | - | cuneatum | 0.0056587 | 0.0015719 | 3.6 | **0.002** |
|  | lancifolium | - | cuneatum | 0.0016682 | 0.0092622 | 0.18 | 1 |
|  | discolor | - | decumbens | 0.0101827 | 0.0018536 | 5.493 | **<0.002** |
|  | lancifolium | - | decumbens | 0.0061922 | 0.009286 | 0.667 | 1 |
|  | lancifolium | - | discolor | -0.0039905 | 0.0092948 | -0.429 | 1 |
| **Diaspore width** | cuneatum | - | catesbaei | -0.3591 | 0.1825 | -1.967 | 0.34 |
|  | decumbens | - | catesbaei | -0.8886 | 0.206 | -4.314 | **<0.001** |
|  | discolor | - | catesbaei | -0.1336 | 0.1015 | -1.316 | 0.94 |
|  | lancifolium | - | catesbaei | -0.4643 | 0.5788 | -0.802 | 1 |
|  | decumbens | - | cuneatum | -0.5295 | 0.1032 | -5.132 | **<0.001** |
|  | discolor | - | cuneatum | 0.2255 | 0.1562 | 1.444 | 0.89 |
|  | lancifolium | - | cuneatum | -0.1053 | 0.5688 | -0.185 | 1 |
|  | discolor | - | decumbens | 0.755 | 0.1836 | 4.111 | **<0.001** |
|  | lancifolium | - | decumbens | 0.4242 | 0.5726 | 0.741 | 1 |
|  | lancifolium | - | discolor | -0.3308 | 0.5739 | -0.576 | 1 |
| **Diaspore length** | cuneatum | - | catesbaei | -0.04863 | 0.16532 | -0.294 | 1 |
|  | decumbens | - | catesbaei | -0.40048 | 0.18681 | -2.144 | 0.29 |
|  | discolor | - | catesbaei | -0.19048 | 0.09057 | -2.103 | 0.29 |
|  | lancifolium | - | catesbaei | -0.37139 | 0.59833 | -0.621 | 1 |
|  | decumbens | - | cuneatum | -0.35185 | 0.09218 | -3.817 | **0.001** |
|  | discolor | - | cuneatum | -0.14186 | 0.14126 | -1.004 | 1 |
|  | lancifolium | - | cuneatum | -0.32276 | 0.59032 | -0.547 | 1 |
|  | discolor | - | decumbens | 0.20999 | 0.16631 | 1.263 | 1 |
|  | lancifolium | - | decumbens | 0.02909 | 0.59331 | 0.049 | 1 |
|  | lancifolium | - | discolor | -0.1809 | 0.59436 | -0.304 | 1 |
| **Elaiosome Length** | cuneatum | - | catesbaei | -0.78961 | 0.2178 | -3.625 | **0.002** |
|  | decumbens | - | catesbaei | -0.90123 | 0.2462 | -3.661 | **0.002** |
|  | discolor | - | catesbaei | -0.51484 | 0.11878 | -4.334 | **<0.001** |
|  | lancifolium | - | catesbaei | -0.69509 | 0.82936 | -0.838 | 1 |
|  | decumbens | - | cuneatum | -0.11162 | 0.12092 | -0.923 | 1 |
|  | discolor | - | cuneatum | 0.27477 | 0.18603 | 1.477 | 0.84 |
|  | lancifolium | - | cuneatum | 0.09452 | 0.8193 | 0.115 | 1 |
|  | discolor | - | decumbens | 0.38639 | 0.21909 | 1.764 | 0.54 |
|  | lancifolium | - | decumbens | 0.20614 | 0.82305 | 0.25 | 1 |
|  | lancifolium | - | discolor | -0.18025 | 0.82438 | -0.219 | 1 |
| **Total FAs** | cuneatum | - | catesbaei | 0.006376 | 0.555193 | 0.011 | 1 |
|  | decumbens | - | catesbaei | -0.131835 | 0.574143 | -0.23 | 1 |
|  | discolor | - | catesbaei | -0.593352 | 0.216414 | -2.742 | 0.05 |
|  | lancifolium | - | catesbaei | -1.189319 | 0.578966 | -2.054 | 0.28 |
|  | decumbens | - | cuneatum | -0.138211 | 0.194939 | -0.709 | 1 |
|  | discolor | - | cuneatum | -0.599728 | 0.555193 | -1.08 | 1 |
|  | lancifolium | - | cuneatum | -1.195695 | 0.260869 | -4.584 | **<0.001** |
|  | discolor | - | decumbens | -0.461517 | 0.574143 | -0.804 | 1 |
|  | lancifolium | - | decumbens | -1.057484 | 0.319823 | -3.306 | **0.009** |
|  | lancifolium | - | discolor | -0.595967 | 0.578966 | -1.029 | 1 |
| **Hexadecanoic acid** | cuneatum | - | catesbaei | -0.09618 | 0.10993 | -0.875 | 1 |
|  | decumbens | - | catesbaei | -0.1689 | 0.11524 | -1.466 | 0.72 |
|  | discolor | - | catesbaei | -0.21142 | 0.05224 | -4.047 | **<0.001** |
|  | lancifolium | - | catesbaei | -0.34022 | 0.11641 | -2.923 | **0.03** |
|  | decumbens | - | cuneatum | -0.07272 | 0.04675 | -1.555 | 0.72 |
|  | discolor | - | cuneatum | -0.11524 | 0.10993 | -1.048 | 1 |
|  | lancifolium | - | cuneatum | -0.24405 | 0.06101 | -4 | **<0.001** |
|  | discolor | - | decumbens | -0.04252 | 0.11524 | -0.369 | 1 |
|  | lancifolium | - | decumbens | -0.17132 | 0.07481 | -2.29 | 0.15 |
|  | lancifolium | - | discolor | -0.1288 | 0.11641 | -1.106 | 1 |
| **Oleic acid** | cuneatum | - | catesbaei | -0.10517 | 0.3148 | -0.334 | 1 |
|  | decumbens | - | catesbaei | -0.18314 | 0.32868 | -0.557 | 1 |
|  | discolor | - | catesbaei | -0.40261 | 0.14187 | -2.838 | 0.05 |
|  | lancifolium | - | catesbaei | -0.62874 | 0.33189 | -1.894 | 0.41 |
|  | decumbens | - | cuneatum | -0.07797 | 0.12722 | -0.613 | 1 |
|  | discolor | - | cuneatum | -0.29744 | 0.3148 | -0.945 | 1 |
|  | lancifolium | - | cuneatum | -0.52356 | 0.16728 | -3.13 | **0.02** |
|  | discolor | - | decumbens | -0.21948 | 0.32868 | -0.668 | 1 |
|  | lancifolium | - | decumbens | -0.4456 | 0.20511 | -2.172 | 0.24 |
|  | lancifolium | - | discolor | -0.22612 | 0.33189 | -0.681 | 1 |
| **Stearic acid** | cuneatum | - | catesbaei | 0.1092 | 0.6234 | 0.175 | 1 |
|  | decumbens | - | catesbaei | -0.1722 | 0.6386 | -0.27 | 1 |
|  | discolor | - | catesbaei | -0.7965 | 0.202 | -3.943 | **<0.001** |
|  | lancifolium | - | catesbaei | -0.9746 | 0.6428 | -1.516 | 0.86 |
|  | decumbens | - | cuneatum | -0.2814 | 0.1827 | -1.54 | 0.86 |
|  | discolor | - | cuneatum | -0.9058 | 0.6234 | -1.453 | 0.86 |
|  | lancifolium | - | cuneatum | -1.0838 | 0.2486 | -4.36 | **<0.001** |
|  | discolor | - | decumbens | -0.6243 | 0.6386 | -0.978 | 1 |
|  | lancifolium | - | decumbens | -0.8024 | 0.3047 | -2.634 | 0.07 |
|  | lancifolium | - | discolor | -0.1781 | 0.6428 | -0.277 | 1 |
| **Linoleic acid** | cuneatum | - | catesbaei | -0.01398 | 0.38576 | -0.036 | 1 |
|  | decumbens | - | catesbaei | -0.18767 | 0.39847 | -0.471 | 1 |
|  | discolor | - | catesbaei | -0.45184 | 0.14748 | -3.064 | **0.02** |
|  | lancifolium | - | catesbaei | -0.89245 | 0.40175 | -2.221 | 0.18 |
|  | decumbens | - | cuneatum | -0.17369 | 0.13291 | -1.307 | 1 |
|  | discolor | - | cuneatum | -0.43786 | 0.38576 | -1.135 | 1 |
|  | lancifolium | - | cuneatum | -0.87847 | 0.17822 | -4.929 | **<0.001** |
|  | discolor | - | decumbens | -0.26417 | 0.39847 | -0.663 | 1 |
|  | lancifolium | - | decumbens | -0.70478 | 0.21849 | -3.226 | **0.01** |
|  | lancifolium | - | discolor | -0.44061 | 0.40175 | -1.097 | 1 |
| **Palmitoleic acid** | cuneatum | - | catesbaei | -0.91227 | 0.24477 | -3.727 | **0.002** |
|  | decumbens | - | catesbaei | -1.10379 | 0.27825 | -3.967 | **<0.001** |
|  | discolor | - | catesbaei | -1.02766 | 0.2796 | -3.675 | **<0.001** |
|  | lancifolium | - | catesbaei | -1.13922 | 0.27829 | -4.094 | **<0.001** |
|  | decumbens | - | cuneatum | -0.19152 | 0.22374 | -0.856 | 1 |
|  | discolor | - | cuneatum | -0.11539 | 0.24477 | -0.471 | 1 |
|  | lancifolium | - | cuneatum | -0.22695 | 0.22669 | -1.001 | 1 |
|  | discolor | - | decumbens | 0.07613 | 0.27825 | 0.274 | 1 |
|  | lancifolium | - | decumbens | -0.03543 | 0.26479 | -0.134 | 1 |
|  | lancifolium | - | discolor | -0.11156 | 0.27829 | -0.401 | 1 |

**Table 2.** Follow-up ANOVAs to RFCMs and follow-up linear models to RFRMs. Single asterisks indicate compounds selected by both the partial RFCM and RFRM or both the full RFCM and RFRM; double asterisks indicate compounds selected by all four RF models. Significant *P*-values are in bold.

| **ANOVAs for selected important compounds (RFCMs)** |  |  |  |  |  |
| --- | --- | --- | --- | --- | --- |
| **Model** | **Compounds** | **Sum Sq.** | **Df** | **F-value** | **P-value** |
| **Partial RFCM** | lactate | 4.53E+14 | 4 | 7.00 | **<0.001** |
|  | thymine | 1.13E+10 | 4 | 6.55 | **<0.001** |
|  | pyroglutamic acid | 3.93E+14 | 4 | 9.44 | **<0.001** |
|  | citraconate | 7.21E+12 | 4 | 5.60 | **0.002** |
|  | adenine | 1.50E+15 | 4 | 9.93 | **<0.001** |
|  | salicylate* | 8.77E+11 | 4 | 6.98 | **<0.001** |
|  | xanthine* | 1353239135 | 4 | 8.61 | **<0.001** |
|  | hydroxyphenylacetate | 1.55E+14 | 4 | 13.34 | **<0.001** |
|  | histidine* | 6.41E+13 | 4 | 6.36 | **0.001** |
|  | indole 3-carboxylate | 8615674602 | 4 | 11.78 | **<0.001** |
|  | 3, 4-dihydroxyphenylacetate* | 1.52E+11 | 4 | 13.10 | **<0.001** |
|  | methylhistidine** | 7.92E+10 | 4 | 9.63 | **<0.001** |
|  | aconitate | 2.28E+14 | 4 | 5.41 | **0.003** |
|  | citrulline** | 5.69E+12 | 4 | 7.93 | **<0.001** |
|  | glucosamine* | 9.07E+11 | 4 | 10.15 | **<0.001** |
|  | hydroxyphenylpyruvate | 2.34E+12 | 4 | 527.87 | **<0.001** |
|  | homocysteic acid | 1.41E+11 | 4 | 17.44 | **<0.001** |
|  | citrate isocitrate | 2.79E+16 | 4 | 4.68 | **0.006** |
|  | creatinol o-phosphate** | 9.31E+09 | 4 | 2.46 | 0.07 |
|  | d-glucarate** | 1.57E+13 | 4 | 18.23 | **<0.001** |
|  | pantothenate* | 3.08E+14 | 4 | 4.38 | **0.008** |
|  | inosine | 2.75E+12 | 4 | 1.85 | 0.15 |
|  | IMP | 9.11E+11 | 4 | 1.15 | 0.35 |
| **Full RFCM** | lactate | 4.53E+14 | 4 | 7.00 | **<0.001** |
|  | pyroglutamic acid | 3.93E+14 | 4 | 9.44 | **<0.001** |
|  | hydroxy 2-methylsuccinate | 6.42E+14 | 4 | 3.96 | **0.01** |
|  | hydroxyphenylacetate | 1.55E+14 | 4 | 13.34 | **<0.001** |
|  | histidine | 6.41E+13 | 4 | 6.36 | **0.001** |
|  | indole 3-carboxylate* | 8615674602 | 4 | 11.78 | **<0.001** |
|  | methylhistidine** | 7.92E+10 | 4 | 9.63 | **<0.001** |
|  | citrulline** | 5.69E+12 | 4 | 7.93 | **<0.001** |
|  | hydroxyphenylpyruvate | 2.34E+12 | 4 | 527.87 | **<0.001** |
|  | homocysteic acid | 1.41E+11 | 4 | 17.44 | **<0.001** |
|  | creatinol o-phosphate** | 9.31E+09 | 4 | 2.46 | 0.07 |
|  | d-glucarate** | 1.57E+13 | 4 | 18.23 | **<0.001** |
|  | pantothenate | 3.08E+14 | 4 | 4.38 | **0.008** |
|  | X846 | 2.42E+13 | 4 | 3.42 | **0.02** |
|  | X1417 | 3.74E+14 | 4 | 3.50 | **0.02** |
|  | X3731 | 1.10E+14 | 4 | 7.60 | **<0.001** |
|  | X4861 | 3.38E+12 | 4 | 2.57 | 0.06 |
|  | X5318 | 9.44E+09 | 4 | 5.40 | 0.003 |
| **Linear Models for selected important compounds (RFRMs)** |  |  |  |  |  |
| **Model** | **Compounds** | **Estimate** | **S.E.** | **t-value** | **P-value** |
| **Partial RFRM** | salicylate * | -2.10E+06 | 7E+05 | -3.15 | **<0.001** |
|  | guanine | 3.02E+05 | 1E+05 | 2.78 | **0.009** |
|  | xanthine * | -96641 | 22624 | -4.27 | **<0.001** |
|  | histidine * | -4.43E+06 | 7E+06 | -0.66 | 0.52 |
|  | uric acid | -1.57E+06 | 1E+06 | -1.52 | 0.14 |
|  | 3, 4-dihydroxyphenylacetate * | 4.58E+05 | 3E+05 | 1.69 | 0.1 |
|  | methylhistidine ** | 6.06E+05 | 2E+05 | 3.28 | **0.003** |
|  | citrulline ** | -5.75E+06 | 2E+06 | -3.64 | **0.001** |
|  | glucosamine * | -2.36E+06 | 6E+05 | -4.10 | **<0.001** |
|  | creatinol o-phosphate ** | -1.58E+05 | 1E+05 | -1.51 | 0.14 |
|  | d-glucarate ** | 8.45E+06 | 2E+06 | 3.74 | **<0.001** |
|  | pantothenate * | -3.65E+07 | 1E+07 | -2.45 | **0.02** |
| **Full RFRM** | salicylate | -6.23E+05 | 6E+05 | -1.04 | 0.31 |
|  | xanthine | -96641 | 22624 | -4.27 | **<0.001** |
|  | indole 3-carboxylate * | -5.75E+06 | 2E+06 | -3.64 | **0.001** |
|  | citrulline ** | -5.75E+06 | 2E+06 | -3.64 | **0.001** |
|  | d-glucarate ** | 8.45E+06 | 2E+06 | 3.74 | **<0.001** |
|  | methylhistidine ** | 3.65E+07 | 1E+07 | 2.45 | **0.02** |
|  | glucosamine | -2.36E+06 | 6E+05 | -4.10 | **<0.001** |
|  | creatinol o-phosphate ** | -1.87E+07 | 2E+07 | -1.15 | 0.26 |
|  | Unknown X1216 | -2.17E+06 | 5E+05 | -3.95 | **<0.001** |
|  | Unknown X2570 | 4.63E+04 | 1E+06 | 0.05 | 0.96 |
|  | Unknown X4619 | -2.24E+06 | 8E+05 | -2.78 | **0.01** |
|  | Unknown X4870 | 2.30E+05 | 2E+05 | 1.32 | 0.2 |
|  | Unknown X4906 | -2.18E+05 | 70176 | -3.10 | **0.004** |
|  | Unknown X6186 | 3.70E+06 | 2E+06 | 2.28 | **0.03** |

**Fig. 1:** Constrained RDA of general elaiosome phytochemical profiles (n = 6 samples per species; n = 30), with relative abundance of 122 known phytochemical compounds and 7,431 unknown features as the response variables and species as the fixed effect (n = 7,552). Ellipses represent the standard deviation of the points around the centroid for each species. Preferred species are indicated by pink, and less-preferred are indicated by blue. The overall model is significant (*P* = 0.03), and explains 15.52% of the variation in the data. Species is a significant predictor of the total phytochemical composition of elaiosomes, including both known and unknown constituents.

**
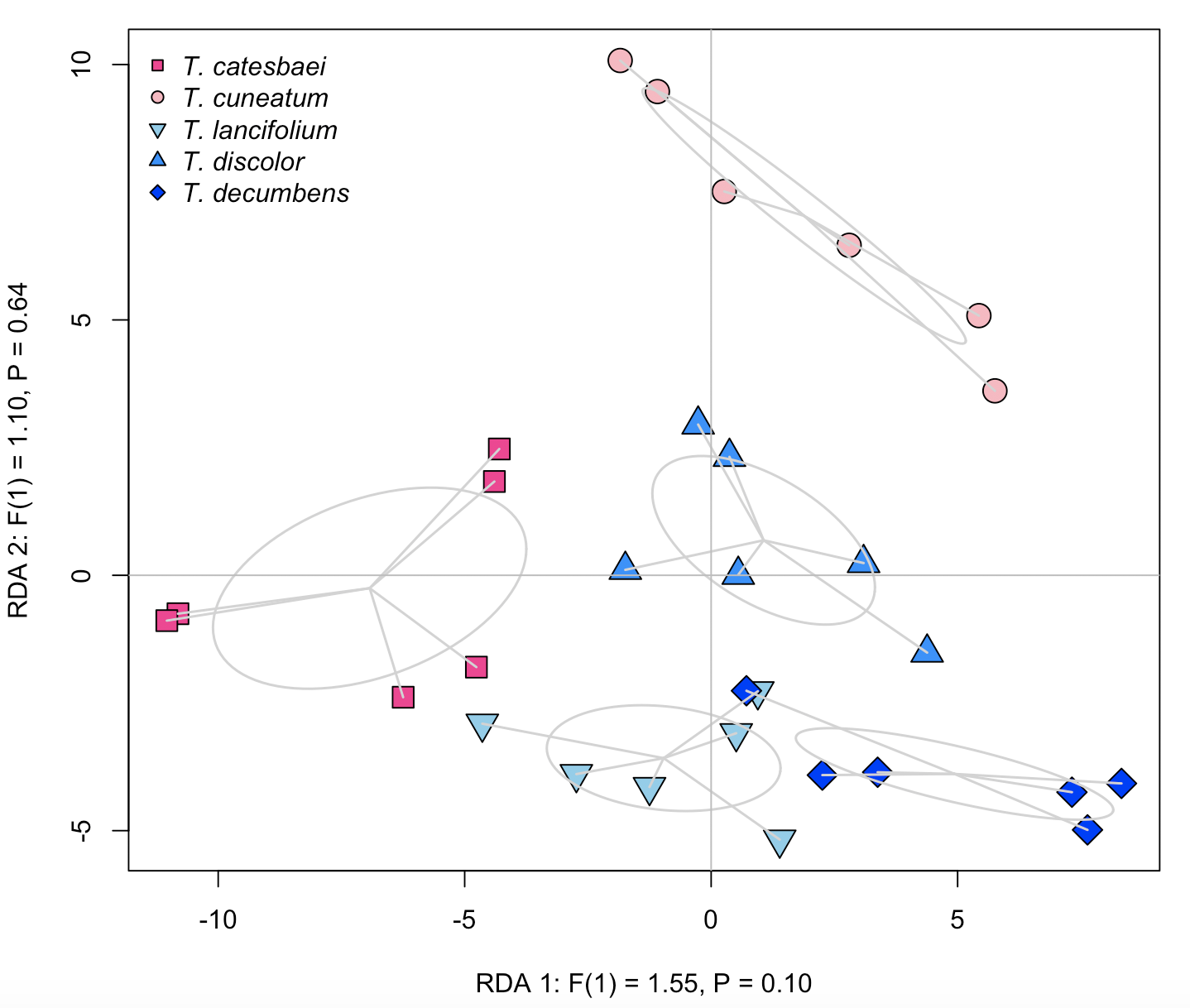
**

**Fig. 2:** Linear relationships between relative concentrations of four elaiosome phytochemicals selected by all random forest models as important in distinguishing among species of *Trillium,* and their corresponding averaged seed dispersal probabilities (0.022 - *T. decumbens,* 0.025 - *T. discolor,* 0.107 - *T. lancifolium*, 0.125 - *T. cuneatum,* 0.168 - *T. catesbaei)*. Equation of the line of best fit is included at the top of each plot. Asterisks indicate significant relationships.

**
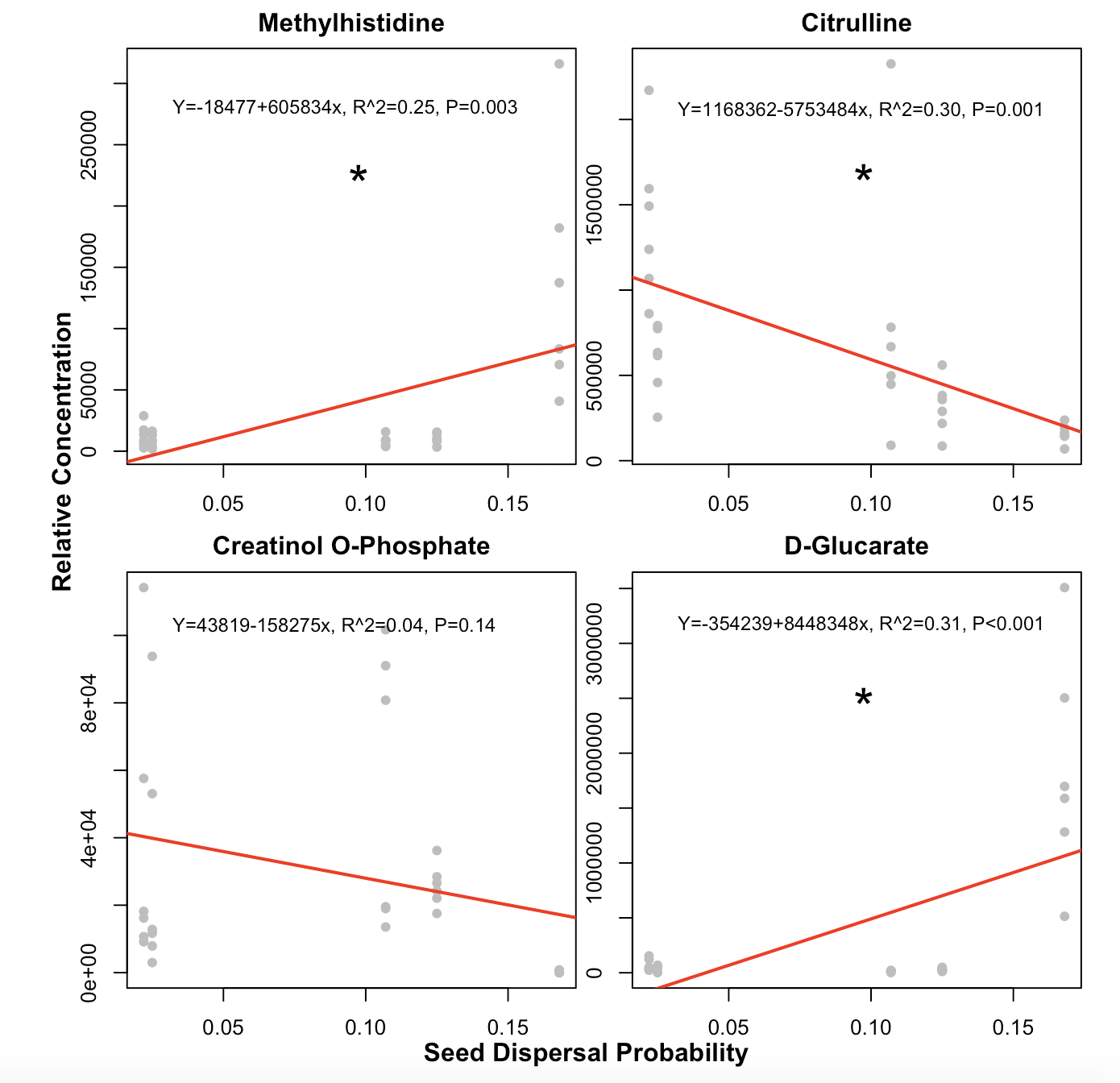
**

**Fig. 3:** Median, interquartile range, and outliers for model residuals depicting the effects of elaiosome fraction (dark grey = diglycerides, light grey = free fatty acids, cream = triglycerides) on concentrations (% fresh weight) for five key fatty acids and total fatty acids sampled from elaiosomes of five species of *Trillium.* Statistical results of linear mixed effects models are included at the bottom left of each plot. Letters indicate Tukey post-hoc pairwise differences.


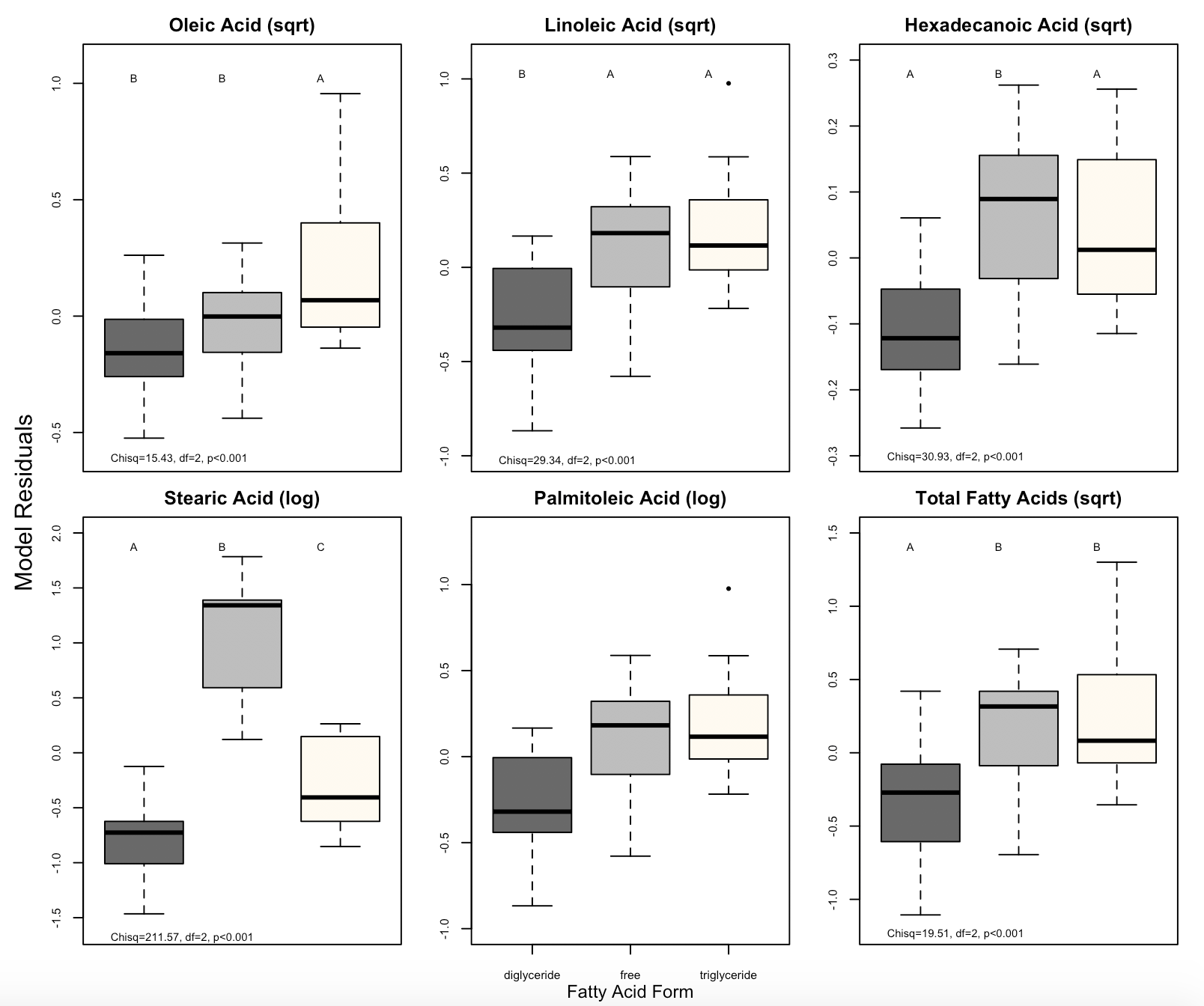


**Fig. 4:** Linear relationships between concentrations (% fresh weight) of total fatty acids (top left), summed free fatty acids (top right), and summed fatty acids in diglycerides (bottom left) and triglycerides (bottom right) among averaged seed dispersal probabilities for the five species of *Trillium* (0.022 - *T. decumbens,* 0.025 - *T. discolor,* 0.107 - *T. lancifolium*, 0.125 - *T. cuneatum,* 0.168 - *T. catesbaei)*. Equation of the line of best fit is included at the top of each plot. Asterisks indicate significant relationships.

**
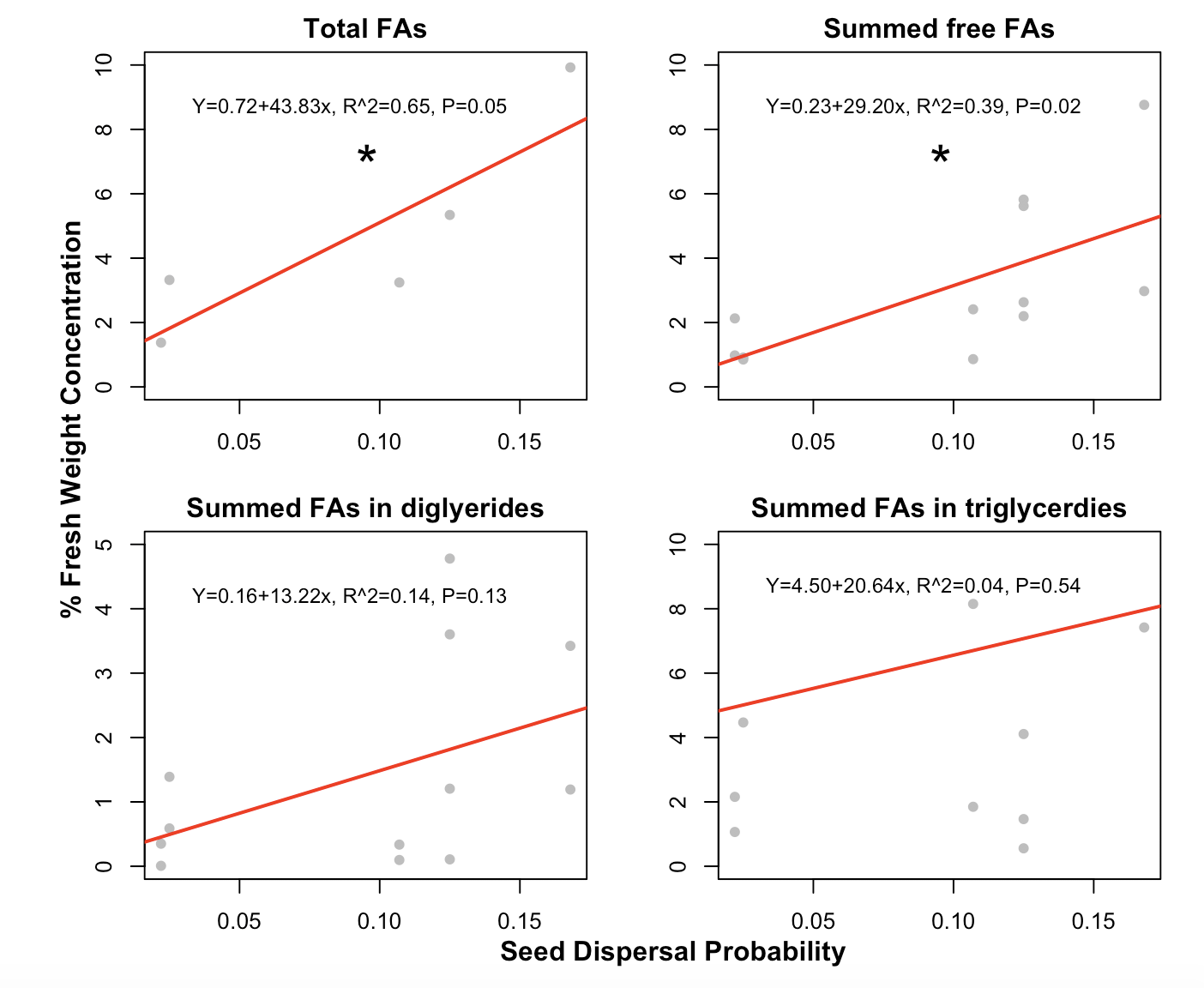
**

**Literature Cited**

Benton, H. P., Want, E. J., & Ebbels, T. D. (2010). Correction of mass calibration gaps in liquid

chromatography-mass spectrometry metabolomics data. Bioinformatics, 26, 2488.

Chambers, M. C., Maclean, B., Burke, R., Amodei, D., Ruderman, D. L., Neumann, S., & …

Hoff, K. (2012). A cross-platform toolkit for mass spectrometry and proteomics. Nature

Biotechnology, 30(10), 918.

Ichihara, K., & Fukubayashi, Y. (2009). Preparation of fatty acid methyl esters for gas-liquid

chromatography. Journal of Lipid Research, 51, 635-640.

Lu, W., Clasquin, M. F., Melamud, E., Amador-Noguez, D. A., Caudy, A., & Rabinowitz, J. D.

(2010). Metabolomic analysis via reversed-phase ion-pairing liquid chromatography

coupled to a stand alone orbitrap mass spectrometer. Analytical Chemistry, 82(8), 3212-

3221.

Smith, C. A., Want, E. J., O'Maille, G., Abagyan, R., & Siuzdak, G. (2006). XCMS: Processing

mass spectrometry data for metabolite profiling using nonlinear peak alignment,

matching and identification. Analytical Chemistry 78: 779–787.

Tautenhahn, R., Boettcher, C., & Neumann, S. (2008). Highly sensitive feature detection for high

resolution LC/MS. BMC Bioinformatics, 9, 504.
